# Vector space algebra for scaling and centering relationship matrices under non-Hardy–Weinberg equilibrium conditions

**DOI:** 10.1186/s12711-020-00589-9

**Published:** 2021-01-18

**Authors:** Luis Gomez-Raya, Wendy M. Rauw, Jack C. M. Dekkers

**Affiliations:** 1 Department of Animal Science, Iowa State University, Ames, IA USA; 2 Dept Mejora Genética Animal, INIA, Madrid, Spain

## Abstract

**Background:**

Scales are linear combinations of variables with coefficients that add up to zero and have a similar meaning to “contrast” in the analysis of variance. Scales are necessary in order to incorporate genomic information into relationship matrices for genomic selection. Statistical and biological parameterizations using scales under different assumptions have been proposed to construct alternative genomic relationship matrices. Except for the natural and orthogonal interactions approach (NOIA) method, current methods to construct relationship matrices assume Hardy–Weinberg equilibrium (HWE). The objective of this paper is to apply vector algebra to center and scale relationship matrices under non-HWE conditions, including orthogonalization by the Gram-Schmidt process.

**Theory and methods:**

Vector space algebra provides an evaluation of current orthogonality between additive and dominance vectors of additive and dominance scales for each marker. Three alternative methods to center and scale additive and dominance relationship matrices based on the Gram-Schmidt process (GSP-A, GSP-D, and GSP-N) are proposed. GSP-A removes additive-dominance co-variation by first fitting the additive and then the dominance scales. GSP-D fits scales in the opposite order. We show that GSP-A is algebraically the same as the NOIA model. GSP-N orthonormalizes the additive and dominance scales that result from GSP-A. An example with genotype information on 32,645 single nucleotide polymorphisms from 903 Large-White × Landrace crossbred pigs is used to construct existing and newly proposed additive and dominance relationship matrices.

**Results:**

An exact test for departures from HWE showed that a majority of loci were not in HWE in crossbred pigs. All methods, except the one that assumes HWE, performed well to attain an average of diagonal elements equal to one and an average of off diagonal elements equal to zero. Variance component estimation for a recorded quantitative phenotype showed that orthogonal methods (NOIA, GSP-A, GSP-N) can adjust for the additive-dominance co-variation when estimating the additive genetic variance, whereas GSP-D does it when estimating dominance components. However, different methods to orthogonalize relationship matrices resulted in different proportions of additive and dominance components of variance.

**Conclusions:**

Vector space methodology can be applied to measure orthogonality between vectors of additive and dominance scales and to construct alternative orthogonal models such as GSP-A, GSP-D and an orthonormal model such as GSP-N. Under non-HWE conditions, GSP-A is algebraically the same as the previously developed NOIA model.

## Background

Currently, massive single nucleotide polymorphism (SNP) genotyping of animals allows genomic prediction [1] with increased accuracy and response to selection compared to pedigree-based prediction of estimated breeding values. A commonly used technique is genomic best linear unbiased prediction (GBLUP) of breeding values, which incorporates a marker-based additive genomic relationship matrix (${\mathbf{G}}$-matrix) instead of a relationship matrix based on pedigree [2]. There are two alternative parameterizations when constructing a genomic relationship matrix: statistical and biological. The statistical or classical parameterization describes breeding values in terms of the average substitution effect of a locus at the population level [3]. The classical parameterization is widely used in breeding value estimation for farm animals because it provides information on the impact of a progenitor on the expected performance of its offspring. The alternative is the biological parameterization in which the effects of a locus are given in terms of genotypic values, which is more intuitive and practical when analyzing variability in natural populations. The distinction between these two alternatives has been previously acknowledged [4] and implemented in the construction of the ${\mathbf{G}}$ matrix for the statistical [2, 5] and biological [6] parameterizations. Most current applications assume that populations are in Hardy–Weinberg equilibrium (HWE). Conditions for HWE are: a very large breeding population, random mating, no change in allele frequencies due to mutation, and absence of migration and selection. The HWE conditions are usually assumed in the construction of the ${\mathbf{G}}$ matrix for simplicity and because departures from those conditions may not be important, in particular if dominance effects are not considered. However, some populations of commercial animals (e.g. pigs and poultry) result from crosses between distant populations, for which HWE does not apply. Recently, Vitezica et al. [7] proposed the construction of genomic relationship matrices with orthogonality between additive and dominance scales based on the NOIA (natural and orthogonal interactions approach) method of Alvarez-Castro and Carlborg [8]. Orthogonality means that additive and dominance effects are uncorrelated. The NOIA method does not require the assumption of HWE. The numerator of ${\mathbf{G}}$ matrices in VanRaden and NOIA are equivalent but the denominators (scaling) are different [9]. Varona et al. [10] reviewed methodology for the construction of relationship matrices that incorporate non-additive effects.

Vector spaces are mathematical objects that abstractly capture the geometry and algebra of linear equations. Some techniques of the algebra of vector spaces, such as the Gram-Schmidt process, have not been applied to the construction of relationship matrices. The objective of this paper is to present and characterize methods to construct orthogonal additive (${\mathbf{G}}$) and dominance (${\mathbf{D}}$) genomic relationship matrices using vector space algebra (Gram-Schmidt process) without requiring HWE. A comparison of the newly proposed methods to construct ${\mathbf{G}}$ and ${\mathbf{D}}$ matrices to existing methods is carried out using a dataset of Large-White $\times$ Landrace crossbred pigs.

## Theory and methods

### Scaling and centering the genomic relationship matrix

In the statistical parameterization, breeding values are modeled using the average of allele substitution effects at genotyped loci, rather than genotypic values. This parameterization requires the assumption of HWE. In 1941, Fisher already pointed out that computation of breeding values must assume random mating [11]. Later and along the same line, Falconer stated: “The concept of breeding value is shown to have no useful meaning when mating is not random” [12]. Therefore, the statistical parameterization will not be considered further and only the biological parameterization will be addressed in the next sections.

The statistical model for genomic prediction with additive and dominance effects in the biological parameterization [6, 13] is:

1 $$y_{i} = \mu + \mathop \sum \limits_{j = 1}^{m} z_{ij} a_{j} + \mathop \sum \limits_{j = 1}^{m} s_{ij} d_{j} + e_{i} ,$$

where $y_{i}$ is the phenotypic record of the $i$-th individual; $\mu$ is the population mean; $m$ is the number of markers; $a_{j}$ and $d_{j}$ are the additive and dominance effects of the $j$-th marker; $z_{ij}$ is 1, 0, and − 1 for the $i$-th individual with genotype at the $j$-th marker $AA$, $Aa$ and $aa$; $s_{ij}$ is 0, 1, and 0 for the $i$-th individual with genotype at the $j$-th marker $AA$, $Aa$ and $aa$; respectively; and $e_{i}$ is the error. In matrix notation, Model (1) is:

$${\mathbf{y}} = \mathbf{1}\mu + {\mathbf{Z}}_{{\mathbf{a}}} {\mathbf{a}} + {\mathbf{S}}_{{\mathbf{d}}} {\mathbf{d}} + {\mathbf{e}},$$

where ${\mathbf{y}}$ is a vector of phenotypes, $\mu$ is the mean; ${\mathbf{Z}}_{{\mathbf{a}}}$ and ${\mathbf{S}}_{{\mathbf{d}}}$ are matrices with $n$ rows (number of individuals) and $m$ columns (number of markers) with values as defined above for $z_{ij}$ and $s_{ij}$ relating information on each individual genotype with additive and dominance effects; ${\mathbf{a}}$ and ${\mathbf{d}}$ are vectors of additive and dominance effects, respectively; and ${\mathbf{e}}$ is a vector of errors. The additive and dominance covariance matrices associated with the random additive and dominance effects in this model are:

$${\text{Cov}}\left( {\mathbf{a}} \right) = \frac{{{\mathbf{H}}_{{\mathbf{a}}} {\mathbf{H}}_{{\mathbf{a}}}^{'} }}{{SC_{a} }}\sigma_{a}^{2} = {\mathbf{G}}\sigma_{a}^{2} ,$$

$${\text{Cov}}\left( {\mathbf{d}} \right) = \frac{{{\mathbf{H}}_{{\mathbf{d}}} {\mathbf{H}}_{{\mathbf{d}}}^{'} }}{{SC_{d} }}\sigma_{d}^{2} = {\mathbf{D}}\sigma_{d}^{2} ,$$

where ${\mathbf{G}}$ and ${\mathbf{D}}$ are additive and dominance genomic relationship matrices; $\sigma_{a}^{2}$ and $\sigma_{d}^{2}$ are the additive and dominance variance, respectively; ${\mathbf{H}}_{{\mathbf{a}}}$ and ${\mathbf{H}}_{{\mathbf{d}}}$ are matrices with $n$ rows and $m$ columns for additive and dominance scales. These scales are used to center all markers so the mean contributed by each marker is zero. $SC_{a}$ and $SC_{d}$ are used for scaling additive and dominance relationship matrices. Different assumptions and methods to center and scale relationships lead to alternative ${\mathbf{G}}$ and ${\mathbf{D}}$ matrices. First, we present several existing models to construct additive and dominance relationships matrices. Second, we propose methods to test for the lack of orthogonality between the additive and dominance scales. Third, we show a general method from vector space algebra, the Gram-Schmidt process, to generate alternative orthogonal models. Last, we present the results obtained by applying these methods to a dataset consisting of crossbred animals.

#### Hardy–Weinberg equilibrium parameterization

Assuming Model (1), the population is segregating for the three genotypes $AA_{\left( j \right)}$, $Aa_{\left( j \right)}$ and $aa_{\left( j \right)}$ at the $j$-th locus, with alleles $A_{\left( j \right)}$ and $a_{\left( j \right)}$ and corresponding frequencies $p_{\left( j \right)}$ and $q_{\left( j \right)}$. The genotypic values under an additive-dominance model of the three genotypes are $a_{\left( j \right)}$, $d_{\left( j \right)}$ and $- a_{\left( j \right)}$, respectively. VanRaden [2] proposed centering of additive marker effects by subtracting the mean additive effect, resulting in the additive genetic values: $\left( {2 - 2p_{\left( j \right)} } \right)a_{\left( j \right)}$, $\left( {1 - 2p_{\left( j \right)} } \right)a_{\left( j \right)}$, $\left( { - 2p_{\left( j \right)} } \right)a_{\left( j \right)}$, for individuals with genotypes $AA_{\left( j \right)}$, $Aa_{\left( j \right)}$ and $aa_{\left( j \right)}$, respectively. Therefore, the scales at the column corresponding to the $j$-th marker in ${\mathbf{H}}_{{\mathbf{a}}}$ are:

2 $$u_{{AA_{\left( j \right)} }}^{hw} = \left( {2 - 2p_{\left( j \right)} } \right), u_{{Aa_{\left( j \right)} }}^{hw} = \left( {1 - 2p_{\left( j \right)} } \right),{\text{ and }}u_{{aa_{\left( j \right)} }}^{hw} = \left( { - 2p_{\left( j \right)} } \right)$$

Assuming HWE, Su et al. [6] and Vitezica et al. [5] proposed centering of dominance marker effects by subtracting the mean dominance effect, resulting in $\left( { - 2p_{\left( j \right)} q_{\left( j \right)} } \right)d_{\left( j \right)}$, $\left( {1 - 2p_{\left( j \right)} q_{\left( j \right)} } \right)d_{\left( j \right)}$, and $\left( { - 2p_{\left( j \right)} q_{\left( j \right)} } \right)d_{\left( j \right)}$ for individuals with genotypes $AA_{\left( j \right)}$, $Aa_{\left( j \right)}$ and $aa_{\left( j \right)}$, respectively. Therefore, the scales at the column corresponding to the $j$-th marker in ${\mathbf{H}}_{{\mathbf{d}}}$ are:

3 $$v_{{AA_{\left( j \right)} }}^{hw} = \left( { - 2p_{\left( j \right)} q_{\left( j \right)} } \right),v_{{Aa_{\left( j \right)} }}^{hw} = \left( {1 - 2p_{\left( j \right)} q_{\left( j \right)} } \right),{\text{ and }}v_{{aa_{\left( j \right)} }}^{hw} = \left( { - 2p_{\left( j \right)} q_{\left( j \right)} } \right)$$

Assuming HWE, VanRaden [2] scaled the additive relationship matrix constructed with scales of Eq. (2) by $\sum\nolimits_{j = 1}^{m} {\left( {2p_{\left( j \right)} q_{\left( j \right)} } \right)}$, while Su et al. [6], and Vitezica et al. [5] proposed scaling the dominance relationship matrix under the assumption of HWE and constructed with scales in Eq. (3) by $\sum\nolimits_{j = 1}^{m} {\left( {2p_{\left( j \right)} q_{\left( j \right)} } \right)\left( {1 - 2p_{\left( j \right)} q_{\left( j \right)} } \right)}$. This approach has two problems. The first one is that it results in functional rather than statistical values associated with locus genotypes, which can be used to derive genotypic values of the individual but not breeding values that predict performance of progeny, as discussed before. Second, centering of the dominance incidence matrix is not necessarily achieved when the population is not in HWE. For example, the mean for the $j$-th marker for the centered dominance matrix is equal to $p_{{AA_{\left( j \right)} }} \left( { - 2p_{\left( j \right)} q_{\left( j \right)} } \right) + p_{{Aa_{\left( j \right)} }} \left( {1 - 2p_{\left( j \right)} q_{\left( j \right)} } \right) + p_{{aa_{\left( j \right)} }} \left( { - 2p_{\left( j \right)} q_{\left( j \right)} } \right)$, where $p_{{AA_{\left( j \right)} }}$, $p_{{Aa_{\left( j \right)} }}$, and $p_{{aa_{\left( j \right)} }}$ are the frequencies of $AA$, $Aa$ and $aa$ genotypes at the $j$-th marker, respectively. It reduces to $p_{{Aa_{\left( j \right)} }} - 2p_{\left( j \right)} q_{\left( j \right)}$ and is not equal to 0 if the population is not in HWE. For example, an *F*_*1*_ cross may have an excess of heterozygotes compared to HWE frequencies.

#### Non-Hardy–Weinberg equilibrium parameterization

Here, we propose a non-HWE parameterization using functional marker effects based on the mean and the variance of additive and dominance genotypic values. Thus, multiplying genotypic frequencies by their corresponding values and summing, the additive mean for the $j$-th marker is:

$$\mu_{{A_{\left( j \right)} }} = a_{\left( j \right)} p_{{AA_{\left( j \right)} }} - a_{\left( j \right)} p_{{aa_{\left( j \right)} }} .$$

Similarly, the variance of the additive genotypic effects is obtained by multiplying genotypic frequencies by the square of their corresponding values, summing, and subtracting the square of the mean:

$$V_{{A_{\left( j \right)} }} = a_{j}^{2} p_{{AA_{\left( j \right)} }} + a_{j}^{2} p_{{aa_{\left( j \right)} }} - \mu_{{A_{\left( j \right)} }}^{2} .$$

After some algebra, the additive variance becomes $V_{{A_{\left( j \right)} }} = a_{j}^{2} \left[ {p_{{AA_{\left( j \right)} }} + p_{{aa_{\left( j \right)} }} - \left( {p_{{AA_{\left( j \right)} }} - p_{{aa_{\left( j \right)} }} } \right)^{2} } \right]$. The additive variance contributed by the $m$ markers is then:

4 $$V_{A} = \mathop \sum \limits_{j = 1}^{m} \left[ {p_{{AA_{\left( j \right)} }} + p_{{aa_{\left( j \right)} }} - \left( {p_{{AA_{\left( j \right)} }} - p_{{aa_{\left( j \right)} }} } \right)^{2} } \right]a_{j}^{2}$$

When subtracting the additive mean from the genotypic values, the centered genotypic values for individuals with genotypes $AA$, $Aa$ and $aa$ at the $j$-th marker in absence of HWE are $\left( {1 - \left( {p_{{AA_{\left( j \right)} }} - p_{{aa_{\left( j \right)} }} } \right)} \right)a_{\left( j \right)} , \left( { - \left( {p_{{AA_{\left( j \right)} }} - p_{{aa_{\left( j \right)} }} } \right)} \right)a_{\left( j \right)}$, and $\left( { - 1 - \left( {p_{{AA_{\left( j \right)} }} - p_{{aa_{\left( j \right)} }} } \right)} \right)a_{\left( j \right)}$. Therefore, in the absence of HWE, the scales at the column corresponding to the $j$-th marker in ${\mathbf{H}}_{{\mathbf{a}}}$ are:

5 $$\begin{aligned} & u_{{AA_{\left( j \right)} }}^{NO - HW} = 1 - \left( {p_{{AA_{\left( j \right)} }} - p_{{aa_{\left( j \right)} }} } \right), \hfill \\ &u_{{Aa_{\left( j \right)} }}^{NO - HW} = - \left( {p_{{AA_{\left( j \right)} }} - p_{{aa_{\left( j \right)} }} } \right),{\text{and}} \hfill \\ &u_{{aa_{\left( j \right)} }}^{NO - HW} = - 1 - \left( {p_{{AA_{\left( j \right)} }} - p_{{aa_{\left( j \right)} }} } \right) \hfill \\ \end{aligned}$$

Note that these centered scales reduce to those of VanRaden [2] because $p_{\left( j \right)} = p_{{AA_{\left( j \right)} }} + \frac{1}{2}p_{{Aa_{\left( j \right)} }}$.

However, the scaling of the additive relationships can be based on Eq. (4), which is different from the classical scaling of matrix ${\mathbf{G}}$ in, e.g., VanRaden [2], which is $\sum\nolimits_{j = 1}^{m} {\left( {2p_{\left( j \right)} q_{\left( j \right)} } \right)}$.

The dominance genotypic effects for the $j$-th marker without the assumption of HWE are as first given by Alvarez-Castro and Carlborg [8]: $- p_{{Aa_{\left( j \right)} }} d_{\left( j \right)}$, $\left( {1 - p_{{Aa_{\left( j \right)} }} } \right)d_{\left( j \right)}$, and $- p_{{Aa_{\left( j \right)} }} d_{\left( j \right)}$ for individuals with genotypes $AA$, $Aa$ and $aa$, respectively. In the absence of HWE, the scales at the column corresponding to the $j$-th marker in ${\mathbf{H}}_{{\mathbf{d}}}$ are:

6 $$v_{{AA_{\left( j \right)} }}^{NO - hw} = - p_{{Aa_{\left( j \right)} }} ,v_{{Aa_{\left( j \right)} }}^{NO - hw} = \left( {1 - p_{{Aa_{\left( j \right)} }} } \right),{\text{ and }}v_{{aa_{\left( j \right)} }}^{NO - hw} = - p_{{Aa_{\left( j \right)} }}$$

The variance contributed by the $j$-th marker is:

$$\begin{aligned} V_{{D_{\left( j \right)} }} =& \left[ {p_{{AA_{\left( j \right)} }} p_{{Aa_{\left( j \right)} }}^{2} + p_{{Aa_{\left( j \right)} }} \left( {1 - p_{{Aa_{\left( j \right)} }} } \right)^{2} + p_{{aa_{\left( j \right)} }} p_{{Aa_{\left( j \right)} }}^{2} } \right]d_{\left( j \right)}^{2} \\ =& \left[ {p_{{Aa_{\left( j \right)} }} - p_{{Aa_{\left( j \right)} }}^{2} } \right]d_{\left( j \right)}^{2} . \\ \end{aligned}$$

Summing over loci, $V_{D} = \sum\nolimits_{j = 1}^{m} {\left[ {p_{{Aa_{\left( j \right)} }} - p_{{Aa_{\left( j \right)} }}^{2} } \right]d_{\left( j \right)}^{2} }$, which renders the scaling of the dominance relationship matrix with $\mathop \sum \limits_{j = 1}^{m} \left[ {p_{{Aa_{\left( j \right)} }} - p_{{Aa_{\left( j \right)} }}^{2} } \right]$. Under HWE, the variance of the dominance effects reduces to $V_{{D_{\left( j \right)} }} = \left[ {2p_{\left( j \right)} q_{\left( j \right)} \left( {1 - 2p_{\left( j \right)} q_{\left( j \right)} } \right)} \right]d_{\left( j \right)}^{2} .$ which is as given by Su et al. [6]. The resulting additive and dominance scales might be correlated (non-orthogonal), which makes estimation and interpretation of the results more difficult.

#### Orthogonal parameterization based on the NOIA method

Based on Cockerham [14], an orthogonal decomposition of additive and dominance variance components was proposed by Alvarez-Castro and Carlborg [8] and termed the natural and orthogonal interactions approach (NOIA). The requirements for the orthogonal partition of variance are:

7 $$\mathop \sum \limits_{j = 1}^{m} p_{{AA_{\left( j \right)} }} u_{{AA_{\left( j \right)} )}}^{NOIA} + p_{{Aa_{\left( j \right)} }} u_{{Aa_{\left( j \right)} }}^{NOIA} + p_{{aa_{\left( j \right)} }} u_{{aa_{\left( j \right)} }}^{NOIA} = 0,$$

8 $$\mathop \sum \limits_{j = 1}^{m} p_{{AA_{\left( j \right)} }} v_{{AA_{\left( j \right)} }}^{NOIA} + p_{{Aa_{\left( j \right)} }} v_{{Aa_{\left( j \right)} }}^{NOIA} + p_{{aa_{\left( j \right)} }} v_{{aa_{\left( j \right)} }}^{NOIA} = 0,$$

9 $$\mathop \sum \limits_{j = 1}^{m} p_{{AA_{\left( j \right)} }} u_{{AA_{\left( j \right)} }}^{NOIA} v_{{AA_{\left( j \right)} }}^{NOIA} + p_{{Aa_{\left( j \right)} }} u_{{Aa_{\left( j \right)} }}^{NOIA}v_{{Aa_{\left( j \right)} }}^{NOIA} + p_{{aa_{\left( j \right)} }} u_{{aa_{\left( j \right)} }}^{NOIA} v_{{aa_{\left( j \right)} }}^{NOIA} = 0,$$

where $u_{{g_{\left( j \right)} }}^{NOIA}$ and $v_{{g_{\left( j \right)} }}^{NOIA}$ are the additive and dominance scales for the $g$-th genotype ($g = AA, Aa, aa$) at the $j$-th marker. Requirements (7) and (8) force a comparison of deviations around the mean for additive and dominance scales. Requirement (9) forces the additive and dominance scales to be uncorrelated (orthogonal).

Vitezica et al. [7] implemented the orthogonalization of NOIA to construct additive and dominance relationships. Thus, the proposed orthogonal scales of Alvarez-Castro and Carlborg [8] and Vitezica et al. [7] for individuals with genotypes $AA$, $Aa$, and $aa$ are:

10 $$\begin{aligned} &u_{{AA_{\left( j \right)} }}^{NOIA} = - \left( { - p_{{Aa_{\left( j \right)} }} - 2p_{{aa_{\left( j \right)} }} } \right), \hfill \\ &u_{{Aa_{\left( j \right)} }}^{NOIA} = - \left( {1 - p_{{Aa_{\left( j \right)} }} - 2p_{{aa_{\left( j \right)} }} } \right), \hfill \\ &u_{{aa_{\left( j \right)} }}^{NOIA} = - \left( {2 - p_{{Aa_{\left( j \right)} }} - 2p_{{aa_{\left( j \right)} }} } \right) \hfill \\ \end{aligned}$$

After some algebra, scales for the additive component in the absence of HWE become the same as for the NOIA method:

$$u_{{AA_{\left( j \right)} }}^{NO - HW} = 1 - \left( {p_{{AA_{\left( j \right)} }} - p_{{aa_{\left( j \right)} }} } \right) = - \left( { - p_{{Aa_{\left( j \right)} }} - 2p_{{aa_{\left( j \right)} }} } \right),$$

$$u_{{Aa_{\left( j \right)} }}^{NO - HW} = - \left( {p_{{AA_{\left( j \right)} }} - p_{{aa_{\left( j \right)} }} } \right) = - \left( {1 - p_{{Aa_{\left( j \right)} }} - 2p_{{aa_{\left( j \right)} }} } \right),$$

$$u_{{aa_{\left( j \right)} }}^{NO - HW} = - 1 - \left( {p_{{AA_{\left( j \right)} }} - p_{{aa_{\left( j \right)} }} } \right) = - \left( {2 - p_{{Aa_{\left( j \right)} }} - 2p_{{aa_{\left( j \right)} }} } \right).$$

They also reduce to the VanRaden scales, as shown by Joshi et al. [9].

The dominance scales in the NOIA method are:

11 $$\begin{aligned} v_{{AA_{\left( j \right)} }}^{NOIA} = \frac{{ - \left( {2p_{{Aa_{\left( j \right)} }} p_{{aa_{\left( j \right)} }} } \right)}}{{\left( {p_{{AA_{\left( j \right)} }} + p_{{aa_{\left( j \right)} }} } \right) - \left( {p_{{AA_{\left( j \right)} }} - p_{{aa_{\left( j \right)} }} } \right)^{2} }}, \hfill \\ v_{{Aa_{\left( j \right)} }}^{NOIA} = \frac{{\left( {4p_{{AA_{\left( j \right)} }} p_{{aa_{\left( j \right)} }} } \right)}}{{\left( {p_{{AA_{\left( j \right)} }} + p_{{aa_{\left( j \right)} }} } \right) - \left( {p_{{AA_{\left( j \right)} }} - p_{{aa_{\left( j \right)} }} } \right)^{2} }}, \hfill \\ v_{{aa_{\left( j \right)} }}^{NOIA} = \frac{{ - \left( {2p_{{AA_{\left( j \right)} }} p_{{Aa_{\left( j \right)} }} } \right)}}{{\left( {p_{{AA_{\left( j \right)} }} + p_{{aa_{\left( j \right)} }} } \right) - \left( {p_{{AA_{\left( j \right)} }} - p_{{aa_{\left( j \right)} }} } \right)^{2} }} \hfill \\ \end{aligned}$$

Equations (10) and (11) satisfy conditions of orthogonality of Eqs. (7), (8), and (9). Vitezica et al. [7] implemented orthogonalization of the NOIA approach by scaling the ${\mathbf{G}}$ and ${\mathbf{D}}$ matrices by $tr\left( {{\mathbf{H}}_{{\mathbf{a}}} {\mathbf{H}}_{{\mathbf{a}}}^{'} } \right)/n$ and $tr\left( {{\mathbf{H}}_{{\mathbf{d}}} {\mathbf{H}}_{{\mathbf{d}}}^{'} } \right)/n$, respectively, where ${\mathbf{H}}_{{\mathbf{a}}}$ and ${\mathbf{H}}_{{\mathbf{d}}}$ include the scales for individuals according to their genotypes in Eqs. (10) and (11), respectively. Therefore, after scaling, genomic and dominance relationship matrices become:

12 $${\mathbf{G}} = \frac{{{\mathbf{H}}_{{\mathbf{a}}} {\mathbf{H}}_{{\mathbf{a}}}^{'} }}{{tr\left( {{\mathbf{H}}_{{\mathbf{a}}} {\mathbf{H}}_{{\mathbf{a}}}^{'} } \right)/n}} ,$$

13 $${\mathbf{D}} = \frac{{{\mathbf{H}}_{{\mathbf{d}}} {\mathbf{H}}_{{\mathbf{d}}}^{'} }}{{tr\left( {{\mathbf{H}}_{{\mathbf{d}}} {\mathbf{H}}_{{\mathbf{d}}}^{'} } \right)/n}}.$$

### Vector space algebra for orthogonal parameterization using the Gram-Schmidt process

We propose to use algebra of vector spaces to construct genomic and dominance relationship matrices. The Gram-Schmidt process takes several non-orthogonal linearly independent functions to construct an orthogonal basis over an arbitrary weighting function [15]. First, we will use vector space algebra to measure orthogonality between the additive and dominance scales.

#### Vectors space algebra to measure orthogonality

Define vector spaces for additive ($$\overset{\lower0.5em\hbox{$\smash{\scriptscriptstyle\rightharpoonup}$}} {u}_{j}$$) and dominance ($$\overset{\lower0.5em\hbox{$\smash{\scriptscriptstyle\rightharpoonup}$}} {v}$$_*j*_) for the $$j$$-th marker with dimensions equal to the number of individuals. The elements of $$\overset{\lower0.5em\hbox{$\smash{\scriptscriptstyle\rightharpoonup}$}} {u}_{j}$$ and $$\overset{\lower0.5em\hbox{$\smash{\scriptscriptstyle\rightharpoonup}$}} {v}$$_*j*_ are the scales to center the additive and dominance relationship matrices, respectively. Then, $${\mathbf{H}}_{{\mathbf{a}}}$$ and $${\mathbf{H}}_{{\mathbf{d}}}$$ can be constructed as:

$${\mathbf{H}}_{{\mathbf{a}}} = \left[ {\overset{\lower0.5em\hbox{$\smash{\scriptscriptstyle\rightharpoonup}$}} {u}_{1} \overset{\lower0.5em\hbox{$\smash{\scriptscriptstyle\rightharpoonup}$}} {u}_{2} \ldots \overset{\lower0.5em\hbox{$\smash{\scriptscriptstyle\rightharpoonup}$}} {u}_{j} \overset{\lower0.5em\hbox{$\smash{\scriptscriptstyle\rightharpoonup}$}}{ u}_{j + 1} \ldots \overset{\lower0.5em\hbox{$\smash{\scriptscriptstyle\rightharpoonup}$}} {u}_{m} } \right],$$

$${\mathbf{H}}_{{\mathbf{d}}} = \left[ {\overset{\lower0.5em\hbox{$\smash{\scriptscriptstyle\rightharpoonup}$}} {v}_{1} \overset{\lower0.5em\hbox{$\smash{\scriptscriptstyle\rightharpoonup}$}} {v}_{2} \ldots \overset{\lower0.5em\hbox{$\smash{\scriptscriptstyle\rightharpoonup}$}} {v}_{j} \overset{\lower0.5em\hbox{$\smash{\scriptscriptstyle\rightharpoonup}$}} {v}_{j + 1} \ldots \overset{\lower0.5em\hbox{$\smash{\scriptscriptstyle\rightharpoonup}$}} {v}_{m} } \right].$$

The vector spaces for the additive scale, $$\overset{\lower0.5em\hbox{$\smash{\scriptscriptstyle\rightharpoonup}$}} {u}$$_***j***_, at the $$j$$-th marker for individuals with genotypes $$AA$$, $$Aa$$ and $$aa$$ under non-HWE conditions based on Eq. (5) are:

$$u_{{AA_{\left( j \right)} }} = 1 - \left( {p_{{AA_{\left( j \right)} }} - p_{{aa_{\left( j \right)} }} } \right)$$

$$u_{{Aa_{\left( j \right)} }} = - \left( {p_{{AA_{\left( j \right)} }} - p_{{aa_{\left( j \right)} }} } \right),$$

$$u_{{aa_{\left( j \right)} }} = - 1 - \left( {p_{{AA_{\left( j \right)} }} - p_{{aa_{\left( j \right)} }} } \right).$$

The elements of $$\overset{\lower0.5em\hbox{$\smash{\scriptscriptstyle\rightharpoonup}$}} {v}$$_*j*_ are dominance scales for individuals with genotypes $$AA$$, $$Aa$$ and *aa* under non-HWE conditions from Eq. (6):

$$v_{{AA_{\left( j \right)} }} = - p_{{Aa_{\left( j \right)} }} ,$$

$$v_{{Aa_{\left( j \right)} }} = \left( {1 - p_{{Aa_{\left( j \right)} }} } \right),$$

$$v_{{aa_{\left( j \right)} }} = - p_{{Aa_{\left( j \right)} }} .$$

For a given marker, the set of vectors, $$\overset{\lower0.5em\hbox{$\smash{\scriptscriptstyle\rightharpoonup}$}} {u}_{j}$$ and $$\overset{\lower0.5em\hbox{$\smash{\scriptscriptstyle\rightharpoonup}$}} {v}_{j}$$, form a basis since both vectors span the vector space and the vectors in the set are linearly independent. However, the set of vectors in this basis is not necessarily orthogonal. In this setting, Alvarez-Castro and Carlborg [8] showed that orthogonality is only achieved when either the two homozygotes are at the same frequency or there is not any heterozygote. The angle, $$\theta_{j}$$, between the two vectors, $$\overset{\lower0.5em\hbox{$\smash{\scriptscriptstyle\rightharpoonup}$}} {u}_{j}$$ and $$\overset{\lower0.5em\hbox{$\smash{\scriptscriptstyle\rightharpoonup}$}} {v}_{j}$$, provides a measure of the degree of orthogonality. From the definition of inner product (p 519 in [15]), $$\cos \theta_{j}$$ for the $$j$$-th marker is given by:

$$\cos \theta_{j} = \frac{{\left\langle {\overset{\lower0.5em\hbox{$\smash{\scriptscriptstyle\rightharpoonup}$}} {u}_{j} , \overset{\lower0.5em\hbox{$\smash{\scriptscriptstyle\rightharpoonup}$}} {v}_{j} } \right\rangle }}{{\left\| {\overset{\lower0.5em\hbox{$\smash{\scriptscriptstyle\rightharpoonup}$}} {u}_{j} } \right\|\left\| {\overset{\lower0.5em\hbox{$\smash{\scriptscriptstyle\rightharpoonup}$}} {v}_{j} } \right\|}},$$

where $$\left\langle {\overset{\lower0.5em\hbox{$\smash{\scriptscriptstyle\rightharpoonup}$}} {u}_{j} , \overset{\lower0.5em\hbox{$\smash{\scriptscriptstyle\rightharpoonup}$}} {v}_{j} } \right\rangle$$ is the inner product of the vectors $$\overset{\lower0.5em\hbox{$\smash{\scriptscriptstyle\rightharpoonup}$}} {u}_{j}$$ and $$\overset{\lower0.5em\hbox{$\smash{\scriptscriptstyle\rightharpoonup}$}} {v}_{j}$$ and has an expected value equal to:

$$\begin{aligned} \left\langle {\overset{\lower0.5em\hbox{$\smash{\scriptscriptstyle\rightharpoonup}$}} {u}_{j} , \overset{\lower0.5em\hbox{$\smash{\scriptscriptstyle\rightharpoonup}$}} {v}_{j} } \right\rangle & = n\left[ \begin{aligned} p_{{AA_{\left( j \right)} }} \left[ {1 - \left( {p_{{AA_{\left( j \right)} }} - p_{{aa_{\left( j \right)} }} } \right)} \right]\left( { - p_{{Aa_{\left( j \right)} }} } \right) - p_{{Aa_{\left( j \right)} }} \left[ {p_{{AA_{\left( j \right)} }} - p_{{aa_{\left( j \right)} }} } \right]\left( {1 - p_{{Aa_{\left( j \right)} }} } \right) \hfill \\ + p_{{aa_{\left( j \right)} }} \left[ { - 1 - \left( {p_{{AA_{\left( j \right)} }} - p_{{aa_{\left( j \right)} }} } \right)} \right]\left( { - p_{{Aa_{\left( j \right)} }} } \right) \hfill \\ \end{aligned} \right] \\ & = n\left( { - p_{{Aa_{\left( j \right)} }} } \right)\left( {p_{{AA_{\left( j \right)} }} - p_{{aa_{\left( j \right)} }} } \right). \\ \end{aligned}$$

and

$$\begin{aligned} \left\| {\overset{\lower0.5em\hbox{$\smash{\scriptscriptstyle\rightharpoonup}$}} {u}_{j} } \right\| & = \sqrt {n\left[ {p_{{AA_{\left( j \right)} }} \left[ {1 - \left( {p_{{AA_{\left( j \right)} }} - p_{{aa_{\left( j \right)} }} } \right)} \right]^{2} + \left[ {p_{{Aa_{\left( j \right)} }} \left[ { - \left( {p_{{AA_{\left( j \right)} }} - p_{{aa_{\left( j \right)} }} } \right)} \right]^{2} + p_{{aa_{\left( j \right)} }} \left[ { - 1 - \left( {p_{{AA_{\left( j \right)} }} - p_{{aa_{\left( j \right)} }} } \right)} \right]^{2} } \right]} \right.} \\ & = \sqrt {n\left[ {p_{{AA_{\left( j \right)} }} + p_{{aa_{\left( j \right)} }} - \left[ {\left( {p_{{AA_{\left( j \right)} }} - p_{{aa_{\left( j \right)} }} } \right)} \right]^{2} } \right]} , \\ \end{aligned}$$

$$\begin{aligned} \left\| {\overset{\lower0.5em\hbox{$\smash{\scriptscriptstyle\rightharpoonup}$}} {v}_{j} } \right\| =& \sqrt {n\left[ {p_{{AA_{\left( j \right)} }} [ - p_{{Aa_{\left( j \right)} }} ]^{2} + p_{{Aa_{\left( j \right)} }} [1 - p_{{Aa_{\left( j \right)} }} ]^{2} + p_{{aa_{\left( j \right)} }} [ - p_{{Aa_{\left( j \right)} }} ]^{2} } \right]} \\ =& \sqrt {n\left[ {p_{{Aa_{\left( j \right)} }} \left[ {1 - p_{{Aa_{\left( j \right)} }} } \right]} \right]} \\ \end{aligned}$$

Taking arc cos in the above formula renders $\theta_{j}$ in radians. For $\theta_{j}$ = 90°, the two vectors are orthogonal. For $\theta_{j}$ ≠ 90°, the two vectors are non-orthogonal, with the level of dependency being larger for values of $\theta_{j}$ near 0° or 180°.

#### Construction of orthogonal ${\mathbf{G}}$ and ${\mathbf{D}}$ matrices using the Gram-Schmidt process

The Gram-Schmidt process can be used to construct an orthogonal basis of the additive and dominance scales for each marker. The basic idea behind orthogonalization by the Gram-Schmidt process is that the first vector is kept unchanged, whereas the common component to both vectors is removed in the second vector. We explored three alternatives for applying the Gram-Schmidt process in the context of genomic relationship matrices. The first, GSP-A, initiates the process with $$\overset{\lower0.5em\hbox{$\smash{\scriptscriptstyle\rightharpoonup}$}} {u}$$ (additive), whereas the second, GSP-D, initiates the process with $$\overset{\lower0.5em\hbox{$\smash{\scriptscriptstyle\rightharpoonup}$}} {v}$$ (dominance). In the third alternative, GSP-N, additive and dominance vector of scales are forced to be of length 1 after using the scales from GSP-A. Conditions of orthogonality from Eqs. (7), (8), and (9) for GSP-A and GSP-D are verified in Appendix 1.

##### GSP-A Gram-Schmidt process

The goal of the process is to obtain orthogonal vectors $$\overset{\lower0.5em\hbox{$\smash{\scriptscriptstyle\rightharpoonup}$}} {\tau }_{A}$$ and $$\overset{\lower0.5em\hbox{$\smash{\scriptscriptstyle\rightharpoonup}$}} {\tau }_{D}$$ for the additive and dominance scales, respectively. The Gram-Schmidt process for GSP-A is:

$$\overset{\lower0.5em\hbox{$\smash{\scriptscriptstyle\rightharpoonup}$}} {\tau }_{{A_{\left( j \right)} }} = \overset{\lower0.5em\hbox{$\smash{\scriptscriptstyle\rightharpoonup}$}} {u}_{\left( j \right)} .$$

Therefore, the elements of $$\overset{\lower0.5em\hbox{$\smash{\scriptscriptstyle\rightharpoonup}$}} {\tau }_{{A_{\left( j \right)} }}$$ are the additive scales from Eq. (5) for the individuals depending on their genotype, $$AA$$, $$Aa$$, or $$aa$$:

14 $$\begin{aligned} &\tau_{{A_{\left( j \right)} }} \left( {AA} \right) = 1 - \left( {p_{{AA_{\left( j \right)} }} - p_{{aa_{\left( j \right)} }} } \right), \hfill \\ &\tau_{{A_{\left( j \right)} }} \left( {Aa} \right) = - \left( {p_{{AA_{\left( j \right)} }} - p_{{aa_{\left( j \right)} }} } \right), \hfill \\ &\tau_{{A_{\left( j \right)} }} \left( {aa} \right) = - 1 - \left( {p_{{AA_{\left( j \right)} }} - p_{{aa_{\left( j \right)} }} } \right) \hfill \\ \end{aligned}$$

For the dominance scales,

$$\overset{\lower0.5em\hbox{$\smash{\scriptscriptstyle\rightharpoonup}$}} {\tau }_{{D_{\left( j \right)} }} = \overset{\lower0.5em\hbox{$\smash{\scriptscriptstyle\rightharpoonup}$}} {v}_{\left( j \right)} - proj_{{\overset{\lower0.5em\hbox{$\smash{\scriptscriptstyle\rightharpoonup}$}} {\tau }_{{A_{\left( j \right)} }} }} \left( {\overset{\lower0.5em\hbox{$\smash{\scriptscriptstyle\rightharpoonup}$}} {v}_{\left( j \right)} } \right),$$

where $$proj_{{\overset{\lower0.5em\hbox{$\smash{\scriptscriptstyle\rightharpoonup}$}} {\tau }_{{A_{\left( j \right)} }} }} \left( {\overset{\lower0.5em\hbox{$\smash{\scriptscriptstyle\rightharpoonup}$}} {v}_{\left( j \right)} } \right) = \frac{{\left\langle {\overset{\lower0.5em\hbox{$\smash{\scriptscriptstyle\rightharpoonup}$}} {\tau }_{{A_{\left( j \right)} }} , \overset{\lower0.5em\hbox{$\smash{\scriptscriptstyle\rightharpoonup}$}} {v}_{\left( j \right)} } \right\rangle }}{{\left\langle {\overset{\lower0.5em\hbox{$\smash{\scriptscriptstyle\rightharpoonup}$}} {\tau }_{{A_{\left( j \right)} }} , \overset{\lower0.5em\hbox{$\smash{\scriptscriptstyle\rightharpoonup}$}} {\tau }_{{A_{\left( j \right)} }} } \right\rangle }}\overset{\lower0.5em\hbox{$\smash{\scriptscriptstyle\rightharpoonup}$}} {\tau }_{{A_{\left( j \right)} }} ,$$,

with $$\left\langle {\overset{\lower0.5em\hbox{$\smash{\scriptscriptstyle\rightharpoonup}$}} {\tau }_{{A_{\left( j \right)} }} , \overset{\lower0.5em\hbox{$\smash{\scriptscriptstyle\rightharpoonup}$}} {v}_{\left( j \right)} } \right\rangle = n\left( { - p_{{Aa_{\left( j \right)} }} } \right)\left( {p_{{AA_{\left( j \right)} }} - p_{{aa_{\left( j \right)} }} } \right)$$

and, $$\left\langle {\overset{\lower0.5em\hbox{$\smash{\scriptscriptstyle\rightharpoonup}$}} {\tau }_{{A_{\left( j \right)} }} , \overset{\lower0.5em\hbox{$\smash{\scriptscriptstyle\rightharpoonup}$}} {\tau }_{{A_{\left( j \right)} }} } \right\rangle = n\left[ {p_{{AA_{\left( j \right)} }} + p_{{aa_{\left( j \right)} }} - \left[ {\left( {p_{{AA_{\left( j \right)} }} - p_{{aa_{\left( j \right)} }} } \right)} \right]^{2} } \right].$$

Substituting $$\left\langle {\overset{\lower0.5em\hbox{$\smash{\scriptscriptstyle\rightharpoonup}$}} {\tau }_{{A_{\left( j \right)} }} , \overset{\lower0.5em\hbox{$\smash{\scriptscriptstyle\rightharpoonup}$}} {v}_{\left( j \right)} } \right\rangle$$ and $$\left\langle {\overset{\lower0.5em\hbox{$\smash{\scriptscriptstyle\rightharpoonup}$}} {\tau }_{{A_{\left( j \right)} }} , \overset{\lower0.5em\hbox{$\smash{\scriptscriptstyle\rightharpoonup}$}} {\tau }_{{A_{\left( j \right)} }} } \right\rangle$$ into the above equation yields:

$$\overset{\lower0.5em\hbox{$\smash{\scriptscriptstyle\rightharpoonup}$}} {\tau }_{{D_{\left( j \right)} }} = \overset{\lower0.5em\hbox{$\smash{\scriptscriptstyle\rightharpoonup}$}} {v}_{\left( j \right)} - \frac{{\left( { - p_{{Aa_{\left( j \right)} }} } \right)\left( {p_{{AA_{\left( j \right)} }} - p_{{aa_{\left( j \right)} }} } \right)}}{{p_{{AA_{\left( j \right)} }} + p_{{aa_{\left( j \right)} }} - \left[ {\left( {p_{{AA_{\left( j \right)} }} - p_{{aa_{\left( j \right)} }} } \right)} \right]^{2} }}\overset{\lower0.5em\hbox{$\smash{\scriptscriptstyle\rightharpoonup}$}} {\tau }_{{A_{\left( j \right)} }} .$$

Therefore, the elements of the orthogonal basis for the dominance vector, $$\overset{\lower0.5em\hbox{$\smash{\scriptscriptstyle\rightharpoonup}$}} {\tau }_{{D_{\left( j \right)} }}$$, for the three genotypes are:

15 $$\begin{aligned} \tau_{{D_{\left( j \right)} }} \left( {AA} \right) = - p_{{Aa_{\left( j \right)} }} - \frac{{ - p_{{Aa_{\left( j \right)} }} \left( {p_{{AA_{\left( j \right)} }} - p_{{aa_{\left( j \right)} }} } \right)}}{{p_{{AA_{\left( j \right)} }} + p_{{aa_{\left( j \right)} }} - \left[ {\left( {p_{{AA_{\left( j \right)} }} - p_{{aa_{\left( j \right)} }} } \right)} \right]^{2} }}\left[ {1 - \left( {p_{{AA_{\left( j \right)} }} - p_{{aa_{\left( j \right)} }} } \right)} \right], \hfill \\ \tau_{{D_{\left( j \right)} }} \left( {Aa} \right) = \left( {1 - p_{{Aa_{\left( j \right)} }} } \right) - \frac{{ - p_{{Aa_{\left( j \right)} }} \left( {p_{{AA_{\left( j \right)} }} - p_{{aa_{\left( j \right)} }} } \right)}}{{p_{{AA_{\left( j \right)} }} + p_{{aa_{\left( j \right)} }} - \left[ {\left( {p_{{AA_{\left( j \right)} }} - p_{{aa_{\left( j \right)} }} } \right)} \right]^{2} }}\left[ { - \left( {p_{{AA_{\left( j \right)} }} - p_{{aa_{\left( j \right)} }} } \right)} \right], \hfill \\ \tau_{{D_{\left( j \right)} }} \left( {aa} \right) = - p_{{Aa_{\left( j \right)} }} - \frac{{ - p_{{Aa_{\left( j \right)} }} \left( {p_{{AA_{\left( j \right)} }} - p_{{aa_{\left( j \right)} }} } \right)}}{{p_{{AA_{\left( j \right)} }} + p_{{aa_{\left( j \right)} }} - \left[ {\left( {p_{{AA_{\left( j \right)} }} - p_{{aa_{\left( j \right)} }} } \right)} \right]^{2} }}\left[ { - 1 - \left( {p_{{AA_{\left( j \right)} }} - p_{{aa_{\left( j \right)} }} } \right)} \right] \hfill \\ \end{aligned}$$

As shown in Appendix 2, the elements for centering the three genotypes from Eq. (15) are algebraically identical to those derived under the NOIA method:

$$\tau_{{D_{\left( j \right)} }} \left( {AA} \right) = \frac{{ - \left( {2p_{{Aa_{\left( j \right)} }} p_{{aa_{\left( j \right)} }} } \right)}}{{\left( {p_{{AA_{\left( j \right)} }} + p_{{aa_{\left( j \right)} }} } \right) - \left( {p_{{AA_{\left( j \right)} }} - p_{{aa_{\left( j \right)} }} } \right)^{2} }},$$

$$\tau_{{D_{\left( j \right)} }} \left( {Aa} \right) = \frac{{\left( {4p_{{AA_{\left( j \right)} }} p_{{aa_{\left( j \right)} }} } \right)}}{{\left( {p_{{AA_{\left( j \right)} }} + p_{{aa_{\left( j \right)} }} } \right) - \left( {p_{{AA_{\left( j \right)} }} - p_{{aa_{\left( j \right)} }} } \right)^{2} }},$$

$$\tau_{{D_{\left( j \right)} }} \left( {aa} \right) = \frac{{ - \left( {2p_{{AA_{\left( j \right)} }} p_{{Aa_{\left( j \right)} }} } \right)}}{{\left( {p_{{AA_{\left( j \right)} }} + p_{{aa_{\left( j \right)} }} } \right) - \left( {p_{{AA_{\left( j \right)} }} - p_{{aa_{\left( j \right)} }} } \right)^{2} }} .$$

The first part of the Gram-Schmidt process leads to an orthogonal basis of additive and dominance scales for each marker. The scaling of additive relationship matrix is as shown in Eq 4. For the dominance component, the variance contributed by the $j$-*th* marker is:

$$\begin{aligned} V_{{D_{\left( j \right)} }} =& p_{{AA_{\left( j \right)} }} \tau_{{D_{\left( j \right)} }}^{2} \left( {AA} \right)d_{\left( j \right)}^{2} + p_{{Aa_{\left( j \right)} }} \tau_{{D_{\left( j \right)} }}^{2} \left( {Aa} \right)d_{\left( j \right)}^{2} + p_{{aa_{\left( j \right)} }} \tau_{{D_{\left( j \right)} }}^{2} \left( {aa} \right)d_{\left( j \right)}^{2} \\ =& \frac{{4p_{{AA_{\left( j \right)} }} p_{{Aa_{\left( j \right)} }} p_{{aa_{\left( j \right)} }} \left( {p_{{Aa_{\left( j \right)} }} p_{{aa_{\left( j \right)} }} + 4p_{{AA_{\left( j \right)} }} p_{{aa_{\left( j \right)} }} + p_{{AA_{\left( j \right)} }} p_{{Aa_{\left( j \right)} }} } \right)}}{{\left[ {p_{{AA_{\left( j \right)} }} + p_{{aa_{\left( j \right)} }} - \left[ {\left( {p_{{AA_{\left( j \right)} }} - p_{{aa_{\left( j \right)} }} } \right)} \right]^{2} } \right]^{2} }}d_{\left( j \right)}^{2} \\ =& \frac{{4p_{{AA_{\left( j \right)} }} p_{{Aa_{\left( j \right)} }} p_{{aa_{\left( j \right)} }} \left[ {4p_{{AA_{\left( j \right)} }} p_{{aa_{\left( j \right)} }} + p_{{AA_{\left( j \right)} }} + p_{{aa_{\left( j \right)} }} - \left( {p_{{AA_{\left( j \right)} }} + p_{{aa_{\left( j \right)} }} } \right)^{2} } \right]}}{{\left[ {p_{{AA_{\left( j \right)} }} + p_{{aa_{\left( j \right)} }} - \left[ {\left( {p_{{AA_{\left( j \right)} }} - p_{{aa_{\left( j \right)} }} } \right)} \right]^{2} } \right]^{2} }}d_{\left( j \right)}^{2} . \\ \end{aligned}$$

After substituting $\left[ {4p_{{AA_{\left( j \right)} }} p_{{aa_{\left( j \right)} }} + p_{{AA_{\left( j \right)} }} + p_{{aa_{\left( j \right)} }} - \left( {p_{{AA_{\left( j \right)} }} + p_{{aa_{\left( j \right)} }} } \right)^{2} } \right]$ by its value: $p_{{AA_{\left( j \right)} }} + p_{{aa_{\left( j \right)} }} - \left[ {\left( {p_{{AA_{\left( j \right)} }} - p_{{aa_{\left( j \right)} }} } \right)} \right]^{2}$, the above equation reduces to:

$$V_{{D_{\left( j \right)} = }} = \frac{{4p_{{AA_{\left( j \right)} }} p_{{Aa_{\left( j \right)} }} p_{{aa_{\left( j \right)} }} }}{{p_{{AA_{\left( j \right)} }} + p_{{aa_{\left( j \right)} }} - \left[ {\left( {p_{{AA_{\left( j \right)} }} - p_{{aa_{\left( j \right)} }} } \right)} \right]^{2} }}d_{\left( j \right)}^{2} .$$

The only difference between GSP-A and the NOIA orthogonalization is in the scaling of the ${\mathbf{G}}$ and ${\mathbf{D}}$ matrices. The scaling in Eqs. (12) and (13) of Vitezica et al. [7] was based on the realized trace of ${\mathbf{H}}_{{\mathbf{a}}} {\mathbf{H}}_{{\mathbf{a}}}^{'}$ or ${\mathbf{H}}_{{\mathbf{d}}} {\mathbf{H}}_{{\mathbf{d}}}^{'}$. In GSP-A, the expected values of those expressions are used for the scaling:

$$E\left[ {\frac{{tr\left( {{\mathbf{H}}_{{\mathbf{a}}} {\mathbf{H}}_{{\mathbf{a}}}^{'} } \right)}}{n}} \right] = \mathop \sum \limits_{j = 1}^{m} \left[ {p_{{AA_{\left( j \right)} }} + p_{{aa_{\left( j \right)} }} - \left( {p_{{AA_{\left( j \right)} }} - p_{{aa_{\left( j \right)} }} } \right)^{2} } \right],$$

$$E\left[ {\frac{{tr\left( {{\mathbf{H}}_{{\mathbf{d}}} {\mathbf{H}}_{{\mathbf{d}}}^{'} } \right)}}{n}} \right] = \mathop \sum \limits_{j = 1}^{m} \left[ {\frac{{4p_{{AA_{\left( j \right)} }} p_{{Aa_{\left( j \right)} }} p_{{aa_{\left( j \right)} }} }}{{p_{{AA_{\left( j \right)} }} + p_{{aa_{\left( j \right)} }} - \left[ {\left( {p_{{AA_{\left( j \right)} }} - p_{{aa_{\left( j \right)} }} } \right)} \right]^{2} }}} \right].$$

For very large $n$, the left and the right-hand sides of the above equations will be the same and also, the relationship matrices constructed using either method.

##### GSP-D Gram-Schmidt process

The Gram-Schmidt process for GSP-D is initiated with the vector for the dominance effects and then the common part of the additive and dominance effects is removed when constructing an orthogonal basis:

$$\overset{\lower0.5em\hbox{$\smash{\scriptscriptstyle\rightharpoonup}$}} {\tau }_{{D_{\left( j \right)} }} = \overset{\lower0.5em\hbox{$\smash{\scriptscriptstyle\rightharpoonup}$}} {v}_{\left( j \right)} .$$

Therefore, dominance scales for the three genotypes in $$\overset{\lower0.5em\hbox{$\smash{\scriptscriptstyle\rightharpoonup}$}} {\tau }_{{D_{\left( j \right)} }}$$ using Eq. 6 are:

16 $$\begin{aligned} &\tau_{{D_{\left( j \right)} }} \left( {AA} \right) = - p_{{Aa_{\left( j \right)} }} , \hfill \\ &\tau_{{D_{\left( j \right)} }} \left( {Aa} \right) = \left( {1 - p_{{Aa_{\left( j \right)} }} } \right), \hfill \\ &\tau_{{D_{\left( j \right)} }} \left( {aa} \right) = - p_{{Aa_{\left( j \right)} }} . \hfill \\ \end{aligned}$$

The scaling for **D** is the same as for the scaling in the non-Hardy–Weinberg parameterization, $\sum\nolimits_{j = 1}^{m} {\left[ {p_{{Aa_{\left( j \right)} }} - p_{{Aa_{\left( j \right)} }}^{2} } \right]}$.

The additive scales are obtained as

17 $$\overset{\lower0.5em\hbox{$\smash{\scriptscriptstyle\rightharpoonup}$}} {\tau }_{{A_{\left( j \right)} }} = \overset{\lower0.5em\hbox{$\smash{\scriptscriptstyle\rightharpoonup}$}} {u}_{\left( j \right)} - proj_{{\overset{\lower0.5em\hbox{$\smash{\scriptscriptstyle\rightharpoonup}$}} {\tau }_{{D_{\left( j \right)} }} }} \left( {\overset{\lower0.5em\hbox{$\smash{\scriptscriptstyle\rightharpoonup}$}} {u}_{\left( j \right)} } \right),$$

where $$proj_{{\overset{\lower0.5em\hbox{$\smash{\scriptscriptstyle\rightharpoonup}$}} {\tau }_{{D_{\left( j \right)} }} }} \left( {\overset{\lower0.5em\hbox{$\smash{\scriptscriptstyle\rightharpoonup}$}} {u}_{\left( j \right)} } \right) = \frac{{\overset{\lower0.5em\hbox{$\smash{\scriptscriptstyle\rightharpoonup}$}} {\tau }_{{D_{\left( j \right)} }} , \overset{\lower0.5em\hbox{$\smash{\scriptscriptstyle\rightharpoonup}$}} {u}_{\left( j \right)} }}{{\overset{\lower0.5em\hbox{$\smash{\scriptscriptstyle\rightharpoonup}$}} {\tau }_{{D_{\left( j \right)} }} , \overset{\lower0.5em\hbox{$\smash{\scriptscriptstyle\rightharpoonup}$}} {\tau }_{{D_{\left( j \right)} }} }}\overset{\lower0.5em\hbox{$\smash{\scriptscriptstyle\rightharpoonup}$}} {\tau }_{{D_{\left( j \right)} }}$$,

with $$\left\langle {\tau_{{D_{\left( j \right)} }} , \overset{\lower0.5em\hbox{$\smash{\scriptscriptstyle\rightharpoonup}$}} {u}_{\left( j \right)} } \right\rangle = n\left( { - p_{{Aa_{\left( j \right)} }} } \right)\left( {p_{{AA_{\left( j \right)} }} - p_{{aa_{\left( j \right)} }} } \right)$$,

and $$\left\langle {\overset{\lower0.5em\hbox{$\smash{\scriptscriptstyle\rightharpoonup}$}} {\tau }_{{D_{\left( j \right)} }} , \overset{\lower0.5em\hbox{$\smash{\scriptscriptstyle\rightharpoonup}$}} {\tau }_{{D_{\left( j \right)} }} } \right\rangle = np_{{Aa_{\left( j \right)} }} \left[ {1 - p_{{Aa_{\left( j \right)} }} } \right].$$

Substituting the above equations into Eq. (17) yields:

$$\overset{\lower0.5em\hbox{$\smash{\scriptscriptstyle\rightharpoonup}$}} {\tau }_{{A_{\left( j \right)} }} = \overset{\lower0.5em\hbox{$\smash{\scriptscriptstyle\rightharpoonup}$}} {u}_{\left( j \right)} - \frac{{\left( { - p_{Aa\left( j \right)} } \right)\left( {p_{AA\left( j \right)} - p_{aa\left( j \right)} } \right)}}{{p_{Aa\left( j \right)} \left[ {1 - p_{Aa\left( j \right)} } \right]}}\overset{\lower0.5em\hbox{$\smash{\scriptscriptstyle\rightharpoonup}$}} {\tau }_{D\left( j \right)} .$$

Therefore, the elements of $$\overset{\lower0.5em\hbox{$\smash{\scriptscriptstyle\rightharpoonup}$}} {\tau }_{{A_{\left( j \right)} }}$$ for the three genotypes are:

18 $$\begin{aligned} \tau_{{A_{\left( j \right)} }} \left( {AA} \right) = \left[ {1 - \left( {p_{{AA_{\left( j \right)} }} - p_{{aa_{\left( j \right)} }} } \right)} \right] - \frac{{ - p_{{Aa_{\left( j \right)} }} \left( {p_{{AA_{\left( j \right)} }} - p_{{aa_{\left( j \right)} }} } \right)}}{{p_{{Aa_{\left( j \right)} }} \left[ {1 - p_{{Aa_{\left( j \right)} }} } \right]}}\left( { - p_{{Aa_{\left( j \right)} }} } \right) \hfill \\ = \left[ {1 - \left( {p_{{AA_{\left( j \right)} }} - p_{{aa_{\left( j \right)} }} } \right)} \right] + \frac{{ - p_{{Aa_{\left( j \right)} }} \left( {p_{{AA_{\left( j \right)} }} - p_{{aa_{\left( j \right)} }} } \right)}}{{\left[ {1 - p_{{Aa_{\left( j \right)} }} } \right]}} = \frac{{2p_{{aa_{\left( j \right)} }} }}{{\left[ {1 - p_{{Aa_{\left( j \right)} }} } \right]}} \hfill \\ \end{aligned}$$

19 $$\begin{aligned} \tau_{{A_{\left( j \right)} }} \left( {Aa} \right) =& \left[ { - \left( {p_{{AA_{\left( j \right)} }} - p_{{aa_{\left( j \right)} }} } \right)} \right] - \frac{{ - p_{{Aa_{\left( j \right)} }} \left( {p_{{AA_{\left( j \right)} }} - p_{{aa_{\left( j \right)} }} } \right)}}{{p_{{Aa_{\left( j \right)} }} \left[ {1 - p_{{Aa_{\left( j \right)} }} } \right]}}\left( {1 - p_{{Aa_{\left( j \right)} }} } \right) \\ =& - \left( {p_{{AA_{\left( j \right)} }} - p_{{aa_{\left( j \right)} }} } \right) + \left( {p_{{AA_{\left( j \right)} }} - p_{{aa_{\left( j \right)} }} } \right) = 0 \\ \end{aligned}$$

20 $$\begin{aligned} \tau_{{A_{\left( j \right)} }} \left( {aa} \right) =& \left[ { - 1 - \left( {p_{{AA_{\left( j \right)} }} - p_{{aa_{\left( j \right)} }} } \right)} \right] - \frac{{ - p_{{Aa_{\left( j \right)} }} \left( {p_{{AA_{\left( j \right)} }} - p_{{aa_{\left( j \right)} }} } \right)}}{{p_{{Aa_{\left( j \right)} }} \left[ {1 - p_{{Aa_{\left( j \right)} }} } \right]}}\left( { - p_{{Aa_{\left( j \right)} }} } \right) \\ =& \left[ { - 1 - \left( {p_{{AA_{\left( j \right)} }} - p_{{aa_{\left( j \right)} }} } \right)} \right] + \frac{{ - p_{{Aa_{\left( j \right)} }} \left( {p_{{AA_{\left( j \right)} }} - p_{{aa_{\left( j \right)} }} } \right)}}{{\left[ {1 - p_{{Aa_{\left( j \right)} }} } \right]}} = \frac{{ - 2p_{{AA_{\left( j \right)} }} }}{{\left[ {1 - p_{{Aa_{\left( j \right)} }} } \right]}} \\ \end{aligned}$$

The additive variance contributed for the *m* markers is:

$$V_{{A_{\left( j \right)} }} = \mathop \sum \limits_{j = 1}^{m} \left[ {\frac{{4p_{{AA_{\left( j \right)} }} p_{{aa_{\left( j \right)} }}^{2} + 4p_{{aa_{\left( j \right)} }} p_{{AA_{\left( j \right)} }}^{2} }}{{\left[ {1 - p_{{Aa_{\left( j \right)} }} } \right]^{2} }}} \right]a_{\left( j \right)}^{2}$$

$$= \mathop \sum \limits_{j = 1}^{m} \left[ {\frac{{4p_{{AA_{\left( j \right)} }} p_{{aa_{\left( j \right)} }} }}{{\left[ {p_{{AA_{\left( j \right)} }} + p_{{aa_{\left( j \right)} }} } \right]}}} \right]a_{\left( j \right)}^{2} .$$

##### GSP-N Gram-Schmidt process

The first step of the GSP-N process leads to an orthogonal basis of additive and dominance scales based on GSP-A. The next and final step of the GSP-N process is to obtain an orthonormal basis for additive and dominance scales by dividing $$\overset{\lower0.5em\hbox{$\smash{\scriptscriptstyle\rightharpoonup}$}} {\tau }_{{A_{\left( j \right)} }}$$ and $$\overset{\lower0.5em\hbox{$\smash{\scriptscriptstyle\rightharpoonup}$}} {\tau }_{{D_{\left( j \right)} }}$$ by the norm of each vector. The length of resulting vectors in GSP-N is unity, which implies that all markers contribute equally when constructing the genomic relationship matrices. Using the scales for $$\overset{\lower0.5em\hbox{$\smash{\scriptscriptstyle\rightharpoonup}$}} {\tau }_{{A_{\left( j \right)} }}$$ and $$\overset{\lower0.5em\hbox{$\smash{\scriptscriptstyle\rightharpoonup}$}} {\tau }_{D\left( j \right)}$$ as described in Eqs. (14) and (15), the norm of the additive and dominance vectors are obtained as follows:

$$\left\| {\overset{\lower0.5em\hbox{$\smash{\scriptscriptstyle\rightharpoonup}$}} {\tau }_{{A_{\left( j \right)} }} } \right\| = \sqrt {n\left[ {p_{{AA_{\left( j \right)} }} + p_{{aa_{\left( j \right)} }} - \left[ {\left( {p_{{AA_{\left( j \right)} }} - p_{{aa_{\left( j \right)} }} } \right)} \right]^{2} } \right]} .$$

$$\begin{aligned} \left\| {\overset{\lower0.5em\hbox{$\smash{\scriptscriptstyle\rightharpoonup}$}} {\tau }_{{D_{\left( j \right)} }} } \right\| =& \sqrt {n\frac{{4p_{{AA_{\left( j \right)} }} p_{{Aa_{\left( j \right)} }}^{2} p_{{aa_{\left( j \right)} }}^{2} + 16p_{{AA_{\left( j \right)} }}^{2} p_{{Aa_{\left( j \right)} }} p_{{aa_{\left( j \right)} }}^{2} + 4p_{{AA_{\left( j \right)} }}^{2} p_{{Aa_{\left( j \right)} }}^{2} p_{{aa_{\left( j \right)} }} }}{{\left[ {p_{{AA_{\left( j \right)} }} + p_{{aa_{\left( j \right)} }} - \left[ {p_{{AA_{\left( j \right)} }} - p_{{aa_{\left( j \right)} }} } \right]^{2} } \right]}}} \\ =& \sqrt {n\frac{{4p_{{AA_{\left( j \right)} }} p_{{Aa_{\left( j \right)} }} p_{{aa_{\left( j \right)} }} }}{{p_{{AA_{\left( j \right)} }} + p_{{aa_{\left( j \right)} }} - \left[ {p_{{AA_{\left( j \right)} }} - p_{{aa_{\left( j \right)} }} } \right]^{2} }}} . \\ \end{aligned}$$

Then, matrices ${\mathbf{H}}_{{\mathbf{a}}}$ and ${\mathbf{H}}_{{\mathbf{d}}}$ become:

$${\mathbf{H}}_{{\mathbf{a}}} = \left[ {\frac{{\overset{\lower0.5em\hbox{$\smash{\scriptscriptstyle\rightharpoonup}$}} {\tau }_{{A_{1} }} }}{{\left\| {\overset{\lower0.5em\hbox{$\smash{\scriptscriptstyle\rightharpoonup}$}} {\tau }_{{A_{1} }} } \right\|}} \frac{{\overset{\lower0.5em\hbox{$\smash{\scriptscriptstyle\rightharpoonup}$}} {\tau }_{{A_{2} }} }}{{\left\| {\overset{\lower0.5em\hbox{$\smash{\scriptscriptstyle\rightharpoonup}$}} {\tau }_{{A_{2} }} } \right\|}} \ldots ..\frac{{\overset{\lower0.5em\hbox{$\smash{\scriptscriptstyle\rightharpoonup}$}} {\tau }_{{A_{j} }} }}{{\left\| {\overset{\lower0.5em\hbox{$\smash{\scriptscriptstyle\rightharpoonup}$}} {\tau }_{{A_{j} }} } \right\|}}\frac{{\overset{\lower0.5em\hbox{$\smash{\scriptscriptstyle\rightharpoonup}$}} {\tau }_{{A_{j + 1} }} }}{{\left\| {\overset{\lower0.5em\hbox{$\smash{\scriptscriptstyle\rightharpoonup}$}} {\tau }_{{A_{j + 1} }} } \right\|}} \ldots .. \frac{{\overset{\lower0.5em\hbox{$\smash{\scriptscriptstyle\rightharpoonup}$}} {\tau }_{{A_{m} }} }}{{\left\| {\overset{\lower0.5em\hbox{$\smash{\scriptscriptstyle\rightharpoonup}$}} {\tau }_{{A_{m} }} } \right\|}}} \right],$$

$$\varvec{H}_{\varvec{d}} = \left[ {\frac{{\overset{\lower0.5em\hbox{$\smash{\scriptscriptstyle\rightharpoonup}$}} {\tau }_{{D_{1} }} }}{{\left\| {\overset{\lower0.5em\hbox{$\smash{\scriptscriptstyle\rightharpoonup}$}} {\tau }_{{D_{1} }} } \right\|}} \frac{{\overset{\lower0.5em\hbox{$\smash{\scriptscriptstyle\rightharpoonup}$}} {\tau }_{{D_{2} }} }}{{\left\| {\overset{\lower0.5em\hbox{$\smash{\scriptscriptstyle\rightharpoonup}$}} {\tau }_{{D_{2} }} } \right\|}} \ldots ..\frac{{\overset{\lower0.5em\hbox{$\smash{\scriptscriptstyle\rightharpoonup}$}} {\tau }_{{D_{j} }} }}{{\left\| {\overset{\lower0.5em\hbox{$\smash{\scriptscriptstyle\rightharpoonup}$}} {\tau }_{{D_{j} }} } \right\|}}\frac{{\overset{\lower0.5em\hbox{$\smash{\scriptscriptstyle\rightharpoonup}$}} {\tau }_{{D_{j + 1} }} }}{{\left\| {\overset{\lower0.5em\hbox{$\smash{\scriptscriptstyle\rightharpoonup}$}} {\tau }_{{D_{j + 1} }} } \right\|}} \ldots .. \frac{{\overset{\lower0.5em\hbox{$\smash{\scriptscriptstyle\rightharpoonup}$}} {\tau }_{{D_{m} }} }}{{\left\| {\overset{\lower0.5em\hbox{$\smash{\scriptscriptstyle\rightharpoonup}$}} {\tau }_{{D_{m} }} } \right\|}}} \right].$$

The resulting relationship matrices still need to be scaled, which can be easily done by dividing ${\mathbf{G}}$ and ${\mathbf{D}}$ by $\left[ {\frac{{tr\left( {{\mathbf{H}}_{{\mathbf{a}}} {\mathbf{H}}_{{\mathbf{a}}}^{'} } \right)}}{n}} \right]$ and $\left[ {\frac{{tr\left( {{\mathbf{H}}_{{\mathbf{d}}} {\mathbf{H}}_{{\mathbf{d}}}^{'} } \right)}}{n}} \right],$ respectively.

### Animals and data

A dataset comprising five trials (PHGC17, PHGC21, PHGC23, PHGC24, and PHGC25) aimed at investigating the genetic basis of resistance to porcine reproductive and respiratory syndrome virus (PRRSV) after natural infection was used for variance component estimation. Right after weaning, 903 Landrace × Large White barrows were moved to farms with a history of PRRSV infections. Blood was drawn weekly and curves of viremia over time were constructed using a LOESS (LOcal regrESSion) function. The LOESS function sets a low-degree polynomial at each point using weighted least squares and gives more weight to observations that are near the point for which response is being estimated and less weight to observations further away. This fitting was necessary to account for the natural variation in the concentration of viremia due to sampling and the methodology used to measure viremia in serum. The area under the curve for viremia for each individual, which will be referred to as viral load (*VL*), was the phenotype used for estimating variance components.

A tissue sample obtained from each pig was used for SNP genotyping. DNA extraction and genotyping was carried out using the Infinium HD Assay Ultra protocol (Illumina Inc.) and the Illumina Porcine SNP60 BeadChip [16]. In total, 32,645 single nucleotide polymorphisms were used to construct relationship matrices. Details are in Gomez-Raya et al. [17].

### Construction of ${\mathbf{G}}$ and ${\mathbf{D}}$ matrices and variance component estimation

An exact test for HWE conditions was carried out for all SNPs in the dataset. This test was performed using the Hardy–Weinberg package in the R language (https://cran.r-project.org/web/packages/HardyWeinberg/HardyWeinberg.pdf).

Construction of the ${\mathbf{G}}$ and ${\mathbf{D}}$ matrices was performed using the six methods described in the previous sections. The Kullback–Leibler divergence [18] was used to quantify the divergence between ${\mathbf{G}}$ and ${\mathbf{D}}$. The Kullback–Leibler divergence [18] from ${\mathbf{Q}}$ to ${\mathbf{P}}$ was computed as:

$$D_{KL } \left( {{\mathbf{P}} \left\| {\mathbf{Q}} \right.} \right) = 0.5 \left[ {trace\left( {{\mathbf{Q}}^{ - 1} {\mathbf{P}}} \right) + \left( {{\varvec{\upmu}}_{{\mathbf{Q}}} - {\varvec{\upmu}}_{{\mathbf{P}}} } \right)^{'} {\mathbf{Q}}^{ - 1} \left( {{\varvec{\upmu}}_{{\mathbf{Q}}} - {\varvec{\upmu}}_{{\mathbf{P}}} } \right) - n + ln\frac{{\left| {\mathbf{Q}} \right|}}{{\left| \varvec{P} \right|}}} \right],$$

where ${\varvec{\upmu}}_{{\mathbf{P}}}$ and ${\varvec{\upmu}}_{{\mathbf{Q}}}$ are vectors of the means of the *n* individuals in the ${\mathbf{P}}$ and ${\mathbf{Q}}$ matrices, respectively. ${\mathbf{P}}$ represents a normal multivariate distribution. Multivariate normal distribution ${\mathbf{Q}}$ represents an approximation to ${\mathbf{P}}$. The Kullback–Leibler divergence is the average difference of the number of bits required for encoding samples of ${\mathbf{P}}$ using a code optimized for ${\mathbf{Q}}$ rather than one optimized for ${\mathbf{P}}$. The unit of Kullback–Leibler divergence is the natural unit of information (nats) [19]. Values of $D_{KL } \left( {{\mathbf{P}}||{\mathbf{Q}}} \right)$ equal to zero means that ${\mathbf{P}}$ and ${\mathbf{Q}}$ are the same distributions. The Kullback–Leibler divergence is not a true metric since it is not symmetrical and it does not obey the triangle inequality. Thus, Kullback–Leibler’s divergence from ${\mathbf{P}}$ to ${\mathbf{Q}}$ is different to divergence from ${\mathbf{Q}}$ to ${\mathbf{P}}$. Asymmetrical $D_{KL } \left( {{\mathbf{P}} ||{\mathbf{Q}}} \right)$ > $D_{KL } \left( {{\mathbf{Q}}||{\mathbf{P}}} \right)$ implies that more information is needed to approximate ${\mathbf{P}}$ with ${\mathbf{Q}}$ than the other way around. In our analysis, ${\mathbf{P}}$ and ${\mathbf{Q}}$ were either two additive or dominance relationship matrices as derived for the six methods investigated in this paper.

The statistical model to analyze *VL* included the fixed effects of the mean and trial. Random variables were the additive and the dominance scales of the biological parameterization, as described in the Theory and methods section. Heritability ($h^{2}$) was estimated as the ratio of the estimate of additive genomic variance over the sum of estimates of the variance of each random component. The proportion of dominance variance ($d^{2}$) was estimated by dividing the estimate of the dominance variance component by the sum of the estimates of all random components in the model. The mixed linear models were fitted using ASReml [20], with ${\mathbf{G}}$ and ${\mathbf{D}}$ matrices as described in the previous sections.

## Results

**Fig. 1:**
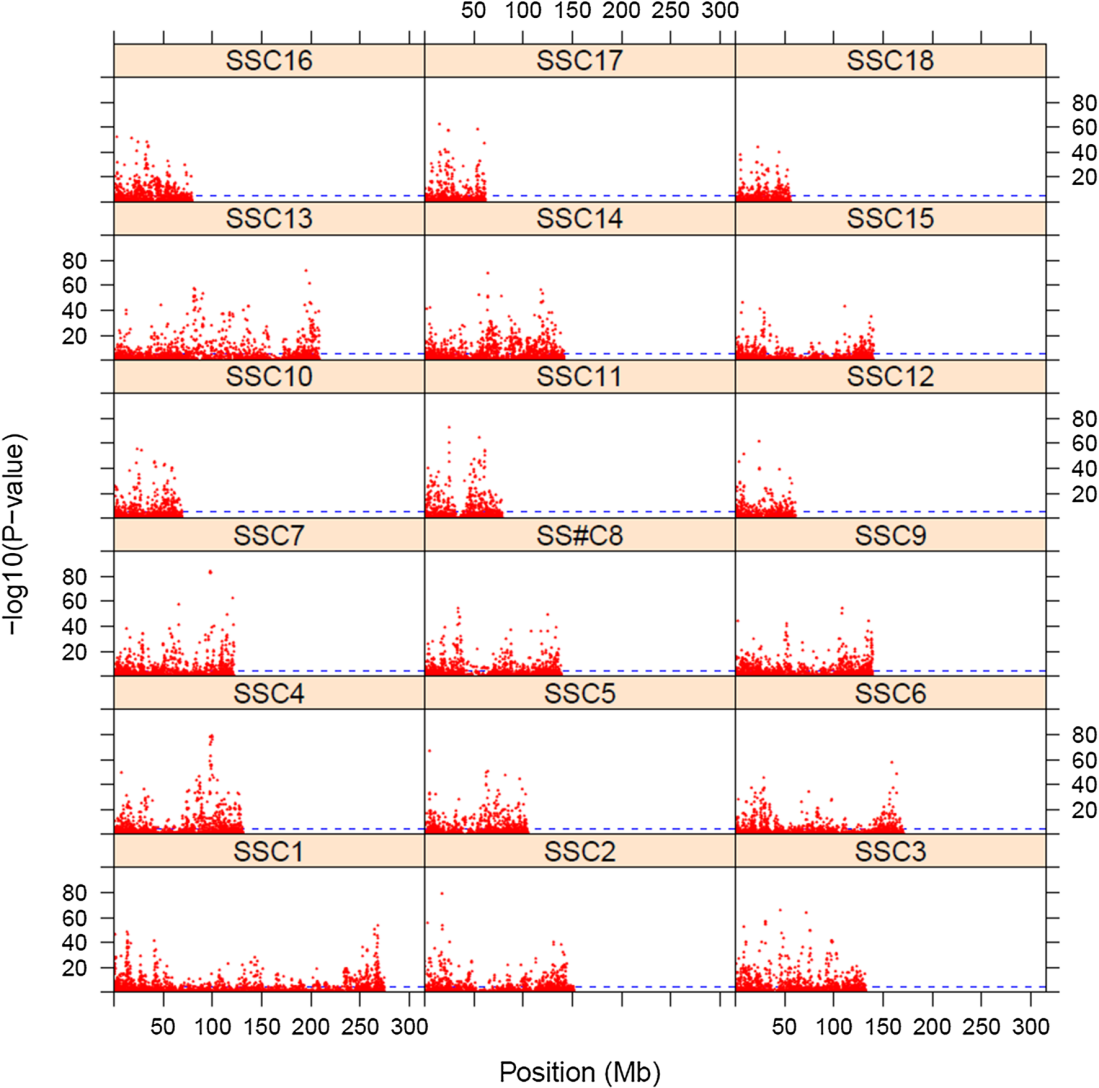
Manhattan plot for departures from Hardy–Weinberg equilibrium in the crossbred population using the Fisher´s exact test

A genome-wide Fisher´s exact test for Hardy–Weinberg departures was performed using all SNPs jointly for all five trials. A Manhattan plot showed that disequilibrium is common in the Landrace × Large-White crosses, although the location of SNPs that were in disequilibrium did not appear to be random (Fig. 1). There was an average excess of 4.7% of heterozygotes across the genome. Thus, these data are appropriate for investigating the properties and comparison of alternative genomic and dominance relationships matrices with departures from HWE.

A first look at the performance of relationship matrices of the six methods revealed that all except the HW approach performed well to attain an average of diagonal elements equal to one and an average off diagonal elements equal to zero (Table 1). The average of the diagonal elements of the ${\mathbf{G}}$ matrix of the HW approach was 0.94 (Table 1). All eigenvalues were positive for all six methods used to construct genomic relationship matrices.

**Table 1:** Average elements of the genomic (${\mathbf{G}}$) and dominance (${\mathbf{D}}$) relationship matrix using biological parameterization when assuming Hardy–Weinberg equilibrium (HW), non-Hardy–Weinberg equilibrium (NO-HW), orthogonal NOIA (NOIA), or the Gram- Schmidt process for additive (GSP-A), dominance (GSP-D), and orthonormal additive (GSP-N) scales

	Relationship matrix					
	HW	NO-HW	NOIA	GSP-A	GSP-D	GSP-N
Average diagonal of ${\mathbf{G}}$	0.942	0.994	1.000	0.999	0.994	1.000
Average off-diagonal of ${\mathbf{G}}$	− 0.001	− 0.001	− 0.001	− 0.001	− 0.001	− 0.001
Range of eigenvalues of ${\mathbf{G}}$	0.000–74.34	0.000–78.45	0.000–78.92	− 0.000 to 78.45	0.001–69.97	0.000–72.17
Average diagonal of ${\mathbf{D}}$	1.003	0.994	1.000	0.994	1.000	1.000
Average off-diagonal of ${\mathbf{D}}$	0.012	− 0.001	− 0.001	− 0.001	− 0.001	− 0.001

Kullback–Leibler’s divergence for pairs of combinations of either ${\mathbf{G}}$ or ${\mathbf{D}}$ matrices is in Table 2. Divergence from ${\mathbf{G}}$ (GSP-D) to ${\mathbf{G}}$ created by other methods was larger than the divergence between other pairs of ${\mathbf{G}}$ matrices. It was also slightly asymmetrical, meaning that more information is needed to approximate ${\mathbf{G}}$ from GSP-D using ${\mathbf{G}}$ from the other methods than the other way around. For the dominance relationship matrices, the Kullback–Leibler divergence using HW relationship distribution is strongly asymmetrical since relationship matrices NOIA, GSP-A, GSP-D, or GSP-N require a much larger number of bits when using a code optimized for HW than the other way around. This could be attributed again to the increase in heterozygosity in the crossbreds, resulting in the scales in ${\mathbf{D}}$ from the HW method not being actually centered to zero. Divergence from ${\mathbf{G}}$ (or ${\mathbf{D}}$) matrices in NOIA and GSP-A methods to ${\mathbf{G}}$ (or ${\mathbf{D}}$) created by other methods were very similar because they are equivalent. The only difference between these two methods is that NOIA uses $tr\left( {{\mathbf{H}}_{{\mathbf{a}}} {\mathbf{H}}_{{\mathbf{a}}}^{'} } \right)/n$ and $tr\left( {{\mathbf{H}}_{{\mathbf{d}}} {\mathbf{H}}_{{\mathbf{d}}}^{'} } \right)/n$ as the denominators of the ${\mathbf{G}}$ and ${\mathbf{D}}$, whereas GSP-A uses just the expected values of these expressions.

**Table 2:** Kullback–Leibler’s divergence for the genomic (${\mathbf{D}}_{{{\mathbf{KL}} }} \left( {{\mathbf{G}}_{1} ||{\mathbf{G}}_{2} } \right))\varvec{ }$ and dominance (${\mathbf{D}}_{{{\mathbf{KL}} }} \left( {{\mathbf{D}}_{1} ||{\mathbf{D}}_{2} } \right))$ relationship matrices based on different parameterizations

${\mathbf{G}}_{2}$	${\mathbf{G}}_{1}$					
	HW	NO-HW	NOIA	GSP-A	GSP-D	GSP-N
HW	0.00	0.90	0.82	0.69	23.58	3.69
NO-HW	0.81	0.00	0.17	0.18	21.71	2.36
NOIA	0.86	0.32	0.00	0.03	21.51	2.50
GSP-A	0.72	0.32	0.03	0.00	21.66	2.54
GSP-D	31.22	26.67	26.11	26.56	0.00	27.88
GSP-N	4.17	2.58	2.61	2.68	24.69	0.00

${\mathbf{D}}_{2}$	${\mathbf{D}}_{1}$					
	HW	NO-HW	NOIA	GSP-A	GSP-D	GSP-N
HW	0.00	1938720.00	928502.90	930729.70	1938720.00	324210.90
NO-HW	7.19	0.00	20.30	20.24	0.00	27.38
NOIA	26.55	19.69	0.00	0.05	19.69	6.17
GSP-A	26.23	19.39	0.05	0.00	19.39	6.13
GSP-D	7.19	0.00	20.30	20.24	0.00	27.38
GSP-N	35.63	30.74	7.03	7.06	30.74	0.00

Values in the upper diagonal are $D_{KL } \left( {G_{1} ||G_{2} } \right))$(or $D_{KL } \left( {D_{1} ||D_{2} } \right))$, while values in the lower diagonal are $D_{KL } \left( {G_{2} ||G_{1} } \right))$(or $D_{KL } \left( {D_{2} ||D_{1} } \right)$). Parametrizations of relationship matrices are: Hardy–Weinberg equilibrium (HW), non-Hardy–Weinberg equilibrium (NO-HW), natural and orthogonal interactions approach (NOIA), and Gram- Schmidt process for additive (GSP-A), dominance (GSP-D) and orthonormal additive (GSP-N)

**Fig. 2:**
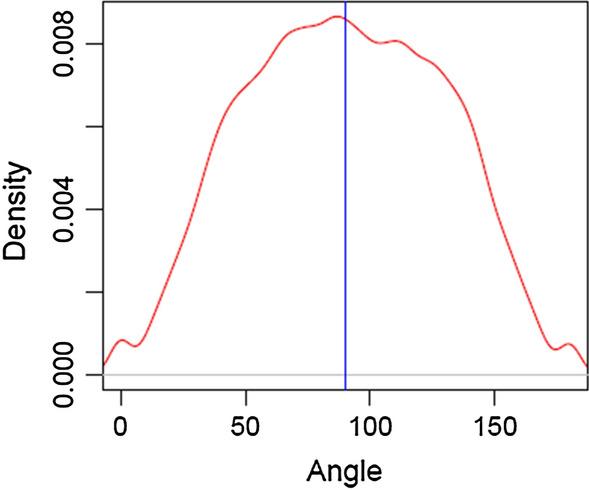
Density of the angle between additive and dominance components across the genome of the crossbred population. The blue vertical bar shows the angle of orthogonality

**Fig. 3:**
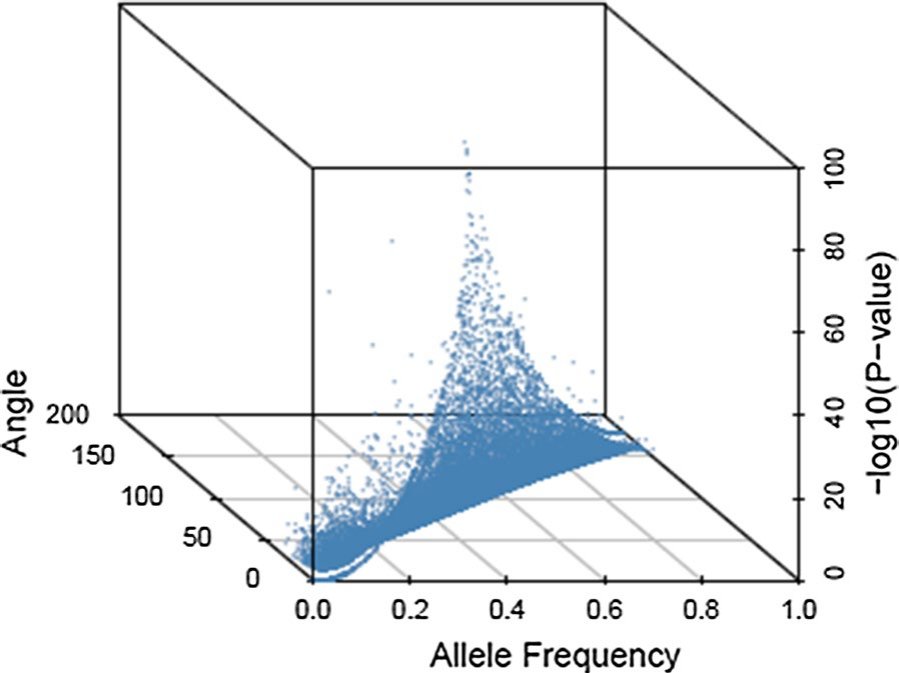
Relationship of the angle between additive and dominance components with allele frequency and with –log10(p-value) of the Fisher´s exact test for departures from Hardy–Weinberg equilibrium

The methodology of the algebra of vector spaces allows to investigate orthogonality between additive and dominance vectors. An angle of 90° between the two vectors implies that the elements of the additive and dominance scales are orthogonal. Figure 2 shows a density plot of the estimates of the angle, $\theta$, between additive and dominance vectors for all markers. Results show that a majority of markers had an angle between the additive and dominance vectors that was close to 90°. In Fig. 3, the –log_10_ (p-value) of Fisher’s exact test for departures from HWE is plotted against the allele frequency and the angle between additive and dominance vectors for each marker when using NO-HW method to construct ${\mathbf{G}}$ and ${\mathbf{D}}$ matrices. Markers with intermediate allele frequencies tended to show significant departures from HWE and tended to have vectors of additive and dominance scales that were orthogonal.

Table 3 shows variance component estimates when using the six alternative methods to construct ${\mathbf{G}}$ and ${\mathbf{D}}$ matrices. The statistical analysis yielded a highly significant trial effect, which is attributable to the uncontrolled conditions in each trial. The NO-HW method tended to have lower estimates of the additive and dominance variance components than HW method, which is consistent with the HW method being upwards biased. Comparing estimates from the orthogonal methods, it can be summarized that (a) NOIA and GSP-A resulted in nearly identical estimates for both additive and dominance variance components, as expected; (b) GSP-D resulted in a larger estimate of the dominance variance component than any of the other methods, which was contrary to estimates from the NOIA and GSP-A methods; and (c) GSP-N resulted in an estimate of zero for dominance variance.

**Table 3:** Estimates of variance components for viral load for the crossbred dataset, using genomic relationship matrices constructed with alternative biological parameterizations: Hardy–Weinberg equilibrium (HW), non-Hardy–Weinberg equilibrium (NO-HW), natural and orthogonal interactions approach (NOIA) and Gram- Schmidt process for additive (GSP-A), dominance (GSP-D) and orthonormal additive (GSP-N)

Parameters	HW	NO-HW	NOIA	GSP-A	GSP-D	GSP-N
$h^{2}$	4.30	4.01	16.08	16.16	0.00	15.86
$d^{2}$	20.35	20.44	1.79	1.80	25.11	0.00
$V_{A}$	58.68	54.63	220.47	221.82	0.0032	217.10
$V_{D}$	277.90	278.31	24.57	24.71	340.86	0.000
$V_{RESIDUAL}$	1029.28	1028.70	1125.84	1125.84	1016.80	1152.07
$V_{PHENOTYPIC}$	1365.86	1361.64	1370.88	1372.37	1357.66	1369.17

$h^{2}$: heritability × 100; $d^{2}$: dominance × 100; heritability and dominance are the additive or dominance variance divided by the sum of all variance components; $V_{A}$: Additive variance; $V_{D}$: dominance variance; $V_{RESIDUAL}$ residual variance; $V_{PHENOTYPIC} = V_{A} + V_{D} + V_{RESIDUAL}$

## Discussion

Most studies in genetics applied to animal breeding or human genetics assume that the populations under study are in HWE. Advances in molecular technologies in recent years have allowed for a renewed interest in the impact of HWE assumptions in genetic analyses [21]. Modern animal breeding now relies on genomic prediction [1]. One of the most widely used methods of genomic prediction is GBLUP, which consists in replacing the traditional pedigree-based relationship matrix by a genomic relationship matrix that incorporates genotype information on SNPs. This requires genotype contributions for each SNP to be centered and scaled for both ${\mathbf{G}}$ and ${\mathbf{D}}$ matrices. The first attempt to construct ${\mathbf{G}}$ matrices from marker genotypes was by VanRaden [2], whose method did not consider dominance and assumed HWE for the scaling of the relationship matrix. Later on, a distinction between genomic relationships constructed using biological and statistical parameterization methods was proposed [5, 6], which also assumed HWE. A new method, NOIA, developed by Alvarez-Castro and Carlborg [8] and applied to the construction of genomic relationship by Vitezica et al. [7] uses an orthogonal partition of additive and dominance effects, and does not require the assumption of HWE. In this paper, we show that vector space algebra can be helpful in the construction of relationships matrices and in the evaluation of the level of departures from orthogonality between additive and dominance vectors of scales. We showed that in our data, vectors of additive and dominance scales constructed using HW-NO are often orthogonal ($\theta$ = 90°). We also show that markers at intermediate frequencies tend to have significant departures from HWE and their vectors of additive and dominant scales are orthogonal. This is because the numerator of cos $\theta_{j}$ is $n\left( { - p_{{Aa_{\left( j \right)} }} } \right)\left( {p_{{AA_{\left( j \right)} }} - p_{{aa_{\left( j \right)} }} } \right)$, which becomes zero (orthogonality) at intermediate frequencies ($p_{{AA_{\left( j \right)} }} = p_{{aa_{\left( j \right)} }}$).

We show in this paper that centering and scaling the ${\mathbf{G}}$ matrix using the NOIA method coincides with the GSP-A method, which is based on orthogonalization by the Gram-Schmidt process. The Gram-Schmidt process converts vectors into an orthogonal system. This is done by taking one of the vectors and finding the projection of the next vector that is orthogonal to the former vector. We also showed that algebraically, GSP-A, and consequently, NOIA only centers and scales by accounting for the lack of HWE. However, orthogonality is achieved in the construction of the ${\mathbf{D}}$ matrix after removing the variation that is common to the ${\mathbf{G}}$ and ${\mathbf{D}}$ matrices. Thus, the proposed applications of the Gram-Schmidt process are equivalent to removing the additive–dominance co-variation from the other relationship matrix by linear regression.

Another alternative to deal with additive-dominance co-variation is inclusion of a covariance term between additive and dominance effects. This was explored by Xiang et al. [22] based on an equivalent statistical model, as proposed by Fernandez et al. [23]. More work is needed to compare this model with models that use NOIA or GSP-A to construct ${\mathbf{G}}$ and ${\mathbf{D}}$ matrices.

One of the most common situations where HWE does not hold is in crossbred populations. Lo et al. [24] first described how to use data on crossbreds and their corresponding purebreds to estimate breeding values and variance components. In their model, each individual has two breeding values; one for purebred performance and one for crossbred performance. Ibañez-Escriche et al. [25] first implemented a crossbred model that incorporates genomic information. More recently, Vitezica et al. [26] showed how additive and dominance components can be implemented in genomic prediction using purebred and crossbred performance to estimate breeding values for purebred animals and their crosses. Our analyses differ from those of Vitezica et al. [26] in that we do not use SNP genotype information on purebreds and just incorporate SNP genotype information from crossbreds into genomic relationship matrices, as an example with extreme departures from HWE (as expected and observed in our analyses). We did not differentiate between allele substitution effects according to the breed origin of the alleles either. In the analysis of crossbred data, the method of Vitezica et al. [26] is more appropriate if the goal is to estimate breeding values of purebreds, and SNP genotype information is available on the purebred parents. Nevertheless, their method also assumes HWE within each of the purebred parental populations, which may affect estimates of variance components.

We observed that roughly between 15 and 25% of all the variation for viral load following PRRSV infection is of genetic origin. Different methods to center and scale relationship matrices provided a different answer to the relative proportion of additive and dominance variation. HW, NO-HW, and GSP-D obtained a much higher estimate of dominance variance than of additive variance, whereas NOIA, GSP-A, and GSP-N resulted in the opposite. This is expected because of the way GSP-A, NOIA, and GSP-N are constructed, i.e. by removing common additive-dominance covariance in the centering of the dominance relationship matrix.

Fisher was the first to separate genetic variance into additive, dominance, and epistatic components using the least squares principle [27]. Later on, Cockerham partitioned the epistatic variance into additive × additive, additive × dominance, dominance × additive, and dominance × dominance interaction components [14]. Cockerham also showed how to scale additive and dominance components using a regression model under the assumption of HWE [14]. He stated “This particular set of scales (among the many others mathematically possible) was chosen for its utility. The scales pertaining to the marginal comparisons of each locus were chosen to separate the marginal variance into the additive (linear) and dominance (quadratic) portions that were long ago shown to be useful for expressing simply the correlation between parent and offspring and between other relatives”. Concerning hybrids, Stuber and Cockerham showed that a proper partitioning can be done in the parental populations, with each being assumed to be in HWE [28]. The NOIA model extends these scales to the situation in which the population is not in HWE [8]. Certainly, NOIA (or GSP-A) reflects better the linear regression nature, for example, of parent–offspring than GSP-D does. Also, it is more appropriate to predict response to selection. However, different scales in GSP-A (or NOIA or GSP-N) versus GSP-D yield different partitions of additive and dominance variance components, which deserves further investigation to address which of the partitions is of interest and for which purpose. In addition, method GSP-N standardized the length of the additive and dominance vectors to one, which implies that all markers weigh equally when constructing genomic relationship matrices, regardless of their frequencies (and/or marker locations). More work is needed to understand the implications and properties of alternative ${\mathbf{G}}$ and ${\mathbf{D}}$ matrices constructed using vector space algebra.

## Conclusions

Vector space theory provides techniques that can be useful for the construction of relationship matrices in populations that are not in HWE. It can provide a measure of the degree of departures from orthogonality between additive and dominance components. It can also be applied to construct orthogonal or orthonormal relationship matrices, such as based on GSP-A, GSP-D, or GSP-N. The GSP-A method coincides with the NOIA method. With the GSP-N method, all markers contribute equally when constructing relationship matrices. Alternative orthogonal models to construct relationship matrices result in different estimates of additive and dominance variances, which requires further research.

## Data Availability

The data that support the findings of this study are accessible from the PRRS Host Genetics Consortium, but restrictions apply to the availability of these data, which were used under license for the current study, and so are not publicly available. Data may be available from the authors upon reasonable request.

## Appendices

### Verification of orthogonalization using the Gram-Schmidt process

In this Appendix, the conditions (7), (8), and (9) for orthogonalization of the Gram-Schmidt process are verified for both GSP-A and GSP-D.

#### Orthogonalization GSP-A

The three requirements for the orthogonal partition of variance components are:

A1 $$\mathop \sum \limits_{j = 1}^{m} p_{{AA_{\left( j \right)} }} \tau_{{D_{\left( j \right)} }} \left( {AA} \right) + p_{{Aa_{\left( j \right)} }} \tau_{{D_{\left( j \right)} }} \left( {Aa} \right) + p_{{aa_{\left( j \right)} }} \tau_{{D_{\left( j \right)} }} \left( {aa} \right) = 0,$$

A2 $$\mathop \sum \limits_{j = 1}^{m} p_{{AA_{\left( j \right)} }} \tau_{{A_{\left( j \right)} }} \left( {AA} \right) + p_{{Aa_{\left( j \right)} }} \tau_{{A_{\left( j \right)} }} \left( {Aa} \right) + p_{{aa_{\left( j \right)} }} \tau_{{A_{\left( j \right)} }} \left( {aa} \right) = 0,$$

A3 $$\mathop \sum \limits_{j = 1}^{m} p_{{AA_{\left( j \right)} }} \tau_{{A_{\left( j \right)} }} \left( {AA} \right)\tau_{{D_{\left( j \right)} }} \left( {AA} \right) + p_{{Aa_{\left( j \right)} }} \tau_{{A_{\left( j \right)} }} \left( {Aa} \right)\tau_{{D_{\left( j \right)} }} \left( {Aa} \right) + p_{{aa_{\left( j \right)} }} \tau_{{A_{\left( j \right)} }} \left( {aa} \right)\tau_{{D_{\left( j \right)} }} \left( {aa} \right) = 0$$

where $m$ is the number of markers; $\tau_{{A_{\left( j \right)} }} \left( k \right)$ and $\tau_{{D_{\left( j \right)} }} \left( k \right)$ are the additive and dominance scales for the $k$-th genotype ($AA$, $Aa$, $aa$) of the $j$-th marker, respectively. Let $\beta_{j} = p_{{AA_{\left( j \right)} }} + p_{{aa_{\left( j \right)} }} - \left[ {\left( {p_{{AA_{\left( j \right)} }} - p_{{aa_{\left( j \right)} }} } \right)} \right]^{2}$. Substituting $\tau_{{D_{\left( j \right)} }} \left( k \right)$ for their values in Eq. (15) into Eq. (A1) yields:

$$\begin{aligned} =& \mathop \sum \limits_{j = 1}^{m} p_{{AA_{\left( j \right)} }} \left[ { - p_{{Aa_{\left( j \right)} }} - \frac{{ - p_{{Aa_{\left( j \right)} }} \left( {p_{{AA_{\left( j \right)} }} - p_{{aa_{\left( j \right)} }} } \right)}}{{\beta_{j} }}\left[ {1 - \left( {p_{{AA_{\left( j \right)} }} - p_{{aa_{\left( j \right)} }} } \right)} \right]} \right] \hfill \\ &\quad + p_{{Aa_{\left( j \right)} }} \left[ {1 - p_{{Aa_{\left( j \right)} }} - \frac{{ - p_{{Aa_{\left( j \right)} }} \left( {p_{{AA_{\left( j \right)} }} - p_{{aa_{\left( j \right)} }} } \right)}}{{\beta_{j} }}\left[ { - \left( {p_{{AA_{\left( j \right)} }} - p_{{aa_{\left( j \right)} }} } \right)} \right]} \right] \hfill \\& \quad + p_{{aa_{\left( j \right)} }} \left[ { - p_{{Aa_{\left( j \right)} }} - \frac{{ - p_{{Aa_{\left( j \right)} }} \left( {p_{{AA_{\left( j \right)} }} - p_{{aa_{\left( j \right)} }} } \right)}}{{\beta_{j} }}\left[ { - 1 - \left( {p_{{AA_{\left( j \right)} }} - p_{{aa_{\left( j \right)} }} } \right)} \right]} \right] \hfill \\ =& \mathop \sum \limits_{j = 1}^{m} - p_{{Aa_{\left( j \right)} }} + p_{{Aa_{\left( j \right)} }} - \frac{{ - p_{{Aa_{\left( j \right)} }} \left( {p_{{AA_{\left( j \right)} }} - p_{{aa_{\left( j \right)} }} } \right)^{2} }}{{\beta_{j} }} + \frac{{ - p_{{Aa_{\left( j \right)} }} \left( {p_{{AA_{\left( j \right)} }} - p_{{aa_{\left( j \right)} }} } \right)^{2} }}{{\beta_{j} }} = 0. \hfill \\ \end{aligned}$$

Substituting $\tau_{{A_{\left( j \right)} }} \left( k \right)$ for their values in Eq. (14) into Eq. (A2) yields:

$$\begin{aligned}& \mathop \sum \limits_{j = 1}^{m} p_{{AA_{\left( j \right)} }} \left[ {1 - \left( {p_{{AA_{\left( j \right)} }} - p_{{aa_{\left( j \right)} }} } \right)} \right] + p_{{Aa_{\left( j \right)} }} \left[ { - \left( {p_{{AA_{\left( j \right)} }} - p_{{aa_{\left( j \right)} }} } \right)} \right] \hfill \\& \quad + p_{{aa_{\left( j \right)} }} \left[ { - 1 - \left( {p_{{AA_{\left( j \right)} }} - p_{{aa_{\left( j \right)} }} } \right)} \right] \hfill \\ =& \mathop \sum \limits_{j = 1}^{m} \left( {p_{{AA_{\left( j \right)} }} - p_{{aa_{\left( j \right)} }} } \right) + \left( {p_{{AA_{\left( j \right)} }} + p_{{Aa_{\left( j \right)} }} + p_{{aa_{\left( j \right)} }} } \right)\left[ { - \left( {p_{{AA_{\left( j \right)} }} - p_{{aa_{\left( j \right)} }} } \right)} \right] = 0. \hfill \\ \end{aligned}$$

Substituting $\tau_{{A_{\left( j \right)} }} \left( k \right)$ and $\tau_{{D_{\left( j \right)} }} \left( k \right)$ for their values in Eqs. (14) and (15) into Eq. (A3) yields:

$$\begin{aligned} &\mathop \sum \limits_{j = 1}^{m} p_{{AA_{\left( j \right)} }} \left[ {1 - \left( {p_{{AA_{\left( j \right)} }} - p_{{aa_{\left( j \right)} }} } \right)} \right]\left[ { - p_{{Aa_{\left( j \right)} }} - \frac{{ - p_{{Aa_{\left( j \right)} )}} \left( {p_{{AA_{\left( j \right)} }} - p_{{aa_{\left( j \right)} }} } \right)}}{{\beta_{j} }}\left[ {1 - \left( {p_{{AA_{\left( j \right)} }} - p_{{aa_{\left( j \right)} }} } \right)} \right]} \right] \hfill \\& \; + p_{{Aa_{\left( j \right)} }} \left[ { - \left( {p_{{AA_{\left( j \right)} }} - p_{{aa_{\left( j \right)} }} } \right)} \right]\left[ {1 - p_{{Aa_{\left( j \right)} }} - \frac{{ - p_{{Aa_{\left( j \right)} }} \left( {p_{{AA_{\left( j \right)} }} - p_{{aa_{\left( j \right)} }} } \right)}}{{\beta_{j} }}\left[ { - \left( {p_{{AA_{\left( j \right)} }} - p_{{aa_{\left( j \right)} }} } \right)} \right]} \right] \hfill \\& \; + p_{{aa_{\left( j \right)} }} \left[ { - 1 - \left( {p_{{AA_{\left( j \right)} }} - p_{{aa_{\left( j \right)} }} } \right)} \right]\left[ { - p_{{Aa_{\left( j \right)} }} - \frac{{ - p_{{Aa_{\left( j \right)} }} \left( {p_{{AA_{\left( j \right)} }} - p_{{aa_{\left( j \right)} }} } \right)}}{{\beta_{j} }}\left[ { - 1 - \left( {p_{{AA_{\left( j \right)} }} - p_{{aa_{\left( j \right)} }} } \right)} \right]} \right]. \hfill \\ \end{aligned}$$

Let

$$\lambda_{j} = - \frac{{ - p_{{Aa_{\left( j \right)} }} \left( {p_{{AA_{\left( j \right)} }} - p_{{aa_{\left( j \right)} }} } \right)}}{{\beta_{j} }}.$$

Then, the above equation becomes:

$$\begin{aligned}& \mathop \sum \limits_{j = 1}^{m} p_{{AA_{\left( j \right)} }} \left[ {1 - \left( {p_{{AA_{\left( j \right)} }} - p_{{aa_{\left( j \right)} }} } \right)} \right]\left[ { - p_{{Aa_{\left( j \right)} }} + \lambda_{j} \left[ {1 - \left( {p_{{AA_{\left( j \right)} }} - p_{{aa_{\left( j \right)} }} } \right)} \right]} \right] \hfill \\& \; + p_{{Aa_{\left( j \right)} }} \left[ { - \left( {p_{{AA_{\left( j \right)} }} - p_{{aa_{\left( j \right)} }} } \right)} \right]\left[ {1 - p_{{Aa_{\left( j \right)} }} + \lambda_{j} \left[ { - \left( {p_{{AA_{\left( j \right)} }} - p_{{aa_{\left( j \right)} }} } \right)} \right]} \right] \hfill \\& \; + p_{{aa_{\left( j \right)} }} \left[ { - 1 - \left( {p_{{AA_{\left( j \right)} }} - p_{aa} } \right)} \right]\left[ { - p_{{Aa_{\left( j \right)} }} + \lambda_{j} \left[ { - 1 - \left( {p_{{AA_{\left( j \right)} }} - p_{{aa_{\left( j \right)} }} } \right)} \right]} \right] \hfill \\ \end{aligned}$$

$$\begin{aligned} =& \mathop \sum \limits_{j = 1}^{m} p_{{AA_{\left( j \right)} }} \left[ { - p_{{Aa_{\left( j \right)} }} + \lambda_{j} \left[ {1 - \left( {p_{{AA_{\left( j \right)} }} - p_{{aa_{\left( j \right)} }} } \right)} \right]} \right] \hfill \\& \; - p_{aa} \left[ { - p_{{Aa_{\left( j \right)} }} + \lambda_{j} } \right[ - 1 - \left( {p_{{AA_{\left( j \right)} }} - p_{{aa_{\left( j \right)} }} } \right)]] \hfill \\& \; + p_{{AA_{\left( j \right)} }} \left[ { - \left( {p_{{AA_{\left( j \right)} }} - p_{{aa_{\left( j \right)} }} } \right)} \right]\left[ { - p_{{Aa_{\left( j \right)} }} + \lambda_{j} } \right[1 - \left( {p_{{AA_{\left( j \right)} }} - p_{{aa_{\left( j \right)} }} } \right)]] \hfill \\& \; + p_{{Aa_{\left( j \right)} }} \left[ { - \left( {p_{{AA_{\left( j \right)} }} - p_{{aa_{\left( j \right)} }} } \right)} \right]\left[ {1 - p_{{Aa_{\left( j \right)} }} + \lambda_{j} } \right[ - \left( {p_{{AA_{\left( j \right)} }} - p_{{aa_{\left( j \right)} }} } \right)]] \hfill \\& \; + p_{{aa_{\left( j \right)} }} \left[ { - \left( {p_{{AA_{\left( j \right)} }} - p_{{aa_{\left( j \right)} }} } \right)} \right]\left[ { - p_{{Aa_{\left( j \right)} }} + \lambda_{j} } \right[ - 1 - \left( {p_{{AA_{\left( j \right)} }} - p_{{aa_{\left( j \right)} }} } \right)]] \hfill \\ \end{aligned}$$

$$\begin{aligned} =& \mathop \sum \limits_{j = 1}^{m} - p_{{Aa_{\left( j \right)} }} \left( {p_{{AA_{\left( j \right)} }} - p_{{aa_{\left( j \right)} }} } \right) + p_{{AA_{\left( j \right)} }} \lambda_{j} \left[ {1 - \left( {p_{{AA_{\left( j \right)} }} - p_{{aa_{\left( j \right)} }} } \right)} \right] \hfill \\& \; - p_{{aa_{\left( j \right)} }} \lambda_{j} \left[ { - 1 - \left( {p_{{AA_{\left( j \right)} }} - p_{{aa_{\left( j \right)} }} } \right)} \right] \hfill \\& \; + p_{{Aa_{\left( j \right)} }} \left( { - \left( {p_{{AA_{\left( j \right)} }} - p_{{aa_{\left( j \right)} }} } \right)} \right) + \left( {p_{{AA_{\left( j \right)} }} + p_{{Aa_{\left( j \right)} }} + p_{{aa_{\left( j \right)} }} } \right)\left( { - p_{{Aa_{\left( j \right)} }} } \right)\left( { - \left( {p_{{AA_{\left( j \right)} }} - p_{{aa_{\left( j \right)} }} } \right)} \right) \hfill \\& \; + \left( {p_{{AA_{\left( j \right)} }} + p_{{Aa_{\left( j \right)} }} + p_{{aa_{\left( j \right)} }} } \right)\left( {p_{{AA_{\left( j \right)} }} - p_{{aa_{\left( j \right)} }} } \right)^{2} \lambda_{j} - \left( {p_{{AA_{\left( j \right)} }} + p_{{Aa_{\left( j \right)} }} + p_{{aa_{\left( j \right)} }} } \right)\left( {p_{{AA_{\left( j \right)} }} - p_{{aa_{\left( j \right)} }} } \right)^{2} \lambda_{j} \hfill \\ \end{aligned}$$

$$= \mathop \sum \limits_{j = 1}^{m} - p_{{Aa_{\left( j \right)} }} \left( {p_{{AA_{\left( j \right)} }} - p_{{aa_{\left( j \right)} }} } \right) + \lambda_{j} \left( {p_{{AA_{\left( j \right)} }} + p_{{aa_{\left( j \right)} }} } \right) - \lambda_{j} \left[ {\left( {p_{{AA_{\left( j \right)} }} - p_{{aa_{\left( j \right)} }} } \right)^{2} } \right]$$

$$\begin{aligned} = \mathop \sum \limits_{j = 1}^{m} - p_{{Aa_{\left( j \right)} }} \left( {p_{{AA_{\left( j \right)} }} - p_{{aa_{\left( j \right)} }} } \right) - \frac{{ - p_{{Aa_{\left( j \right)} }} \left( {p_{{AA_{\left( j \right)} }} - p_{{aa_{\left( j \right)} }} } \right)}}{{\beta_{j} }}\left[ {p_{{AA_{\left( j \right)} }} + p_{{aa_{\left( j \right)} }} - \left( {p_{{AA_{\left( j \right)} }} - p_{{aa_{\left( j \right)} }} } \right)^{2} } \right] \hfill \\ = \mathop \sum \limits_{j = 1}^{m} - p_{{Aa_{\left( j \right)} }} \left( {p_{{AA_{\left( j \right)} }} - p_{{aa_{\left( j \right)} }} } \right) + p_{{Aa_{\left( j \right)} }} \left( {p_{{AA_{\left( j \right)} }} - p_{{aa_{\left( j \right)} }} } \right) = 0. \hfill \\ \end{aligned}$$

#### Orthogonalization GSP-D

Substituting $\tau_{{D_{\left( j \right)} }} \left( k \right)$ for their values in Eq. (16) into Eq. (A1) yields:

$$\begin{aligned} \mathop \sum \limits_{j = 1}^{m} p_{{AA_{\left( j \right)} }} \left( { - p_{{Aa_{\left( j \right)} }} } \right) + p_{{Aa_{\left( j \right)} }} \left( {1 - p_{{Aa_{\left( j \right)} }} } \right) + p_{{aa_{\left( j \right)} }} \left( { - p_{{Aa_{\left( j \right)} }} } \right) \hfill \\ \; = \mathop \sum \limits_{j = 1}^{m} - p_{{Aa_{\left( j \right)} }} \left( {p_{{AA_{\left( j \right)} }} + p_{{Aa_{\left( j \right)} }} + p_{{aa_{\left( j \right)} }} } \right) + p_{{Aa_{\left( j \right)} }} = 0. \hfill \\ \end{aligned}$$

Substituting $\tau_{{A_{\left( j \right)} }} \left( k \right)$ for their values in Eqs. (18), (19) and (20) into Eq. A2 yields:

$$\mathop \sum \limits_{j = 1}^{m} p_{{AA_{\left( j \right)} }} \frac{{2p_{{aa_{\left( j \right)} }} }}{{p_{{AA_{\left( j \right)} }} + p_{{aa_{\left( j \right)} }} }} + p_{{aa_{\left( j \right)} }} \frac{{ - 2p_{{AA_{\left( j \right)} }} }}{{p_{{AA_{\left( j \right)} }} + p_{{aa_{\left( j \right)} }} }} = 0.$$

Substituting $\tau_{{A_{\left( j \right)} }} \left( k \right)$ and $\tau_{{D_{\left( j \right)} }} \left( k \right)$ for their values in Eqs. (6), (18), (19) and (20) into Eq. (A3) yields:

$$\mathop \sum \limits_{j = 1}^{m} p_{{AA_{\left( j \right)} }} \frac{{2p_{{aa_{\left( j \right)} }} }}{{p_{{AA_{\left( j \right)} }} + p_{{aa_{\left( j \right)} }} }}\left( { - p_{{Aa_{\left( j \right)} }} } \right) + p_{{aa_{\left( j \right)} }} \frac{{ - 2p_{{AA_{\left( j \right)} }} }}{{p_{{AA_{\left( j \right)} }} + p_{{aa_{\left( j \right)} }} }}\left( { - p_{{Aa_{\left( j \right)} }} } \right) = 0.$$

### Equivalence of NOIA and GSP-A

In this appendix, the equivalence for centering between the NOIA and GSP-A is shown. This will be accomplished by reducing the formulae obtained by the Gram-Schmidt process to the formulae of NOIA method for each of the three genotypes:

#### Genotypes $\varvec{AA}$

Let $$\beta = \tau_{A} , \tau_{A} = p_{AA} + p_{aa} - \left[ {\left( {p_{AA} - p_{aa} } \right)} \right]^{2}$$,

Equation (15) for individuals with genotypes $AA$ becomes:

$$\begin{aligned} \tau_{D} \left( {AA} \right) =& - p_{Aa} - \frac{{ - p_{Aa} \left( {p_{AA} - p_{aa} } \right)}}{\beta }\left[ {1 - \left( {p_{AA} - p_{aa} } \right)} \right] \\ =& - p_{Aa} + \frac{{p_{Aa} \left[ {\left( {p_{AA} - p_{aa} } \right) - \left( {p_{AA} - p_{aa} } \right)^{2} } \right]}}{\beta } \\ =& - p_{Aa} + \frac{{p_{Aa} \left[ {\left( {p_{AA} + p_{aa} } \right) - \left( {p_{AA} - p_{aa} } \right)^{2} - 2p_{aa} } \right]}}{\beta } \\ =& - p_{Aa} + \frac{{p_{Aa} \left[ {\beta - 2p_{aa} } \right]}}{\beta } \\ =& \frac{{ - p_{Aa} \beta + p_{Aa} \left[ {\beta - 2p_{aa} } \right]}}{\beta } \\ =& \frac{{ - 2p_{Aa} p_{aa} }}{{p_{AA} + p_{aa} - \left[ {\left( {p_{AA} - p_{aa} } \right)} \right]^{2 } }}, \\ \end{aligned}$$

which is the same Eq. (11) for genotypes $AA$ as in the NOIA method.

#### Genotypes $\varvec{Aa}$

Equation (15) for individuals with genotypes $Aa$ becomes:

$$\begin{aligned} \tau_{D} \left( {Aa} \right) =& \left( {1 - p_{Aa} } \right) - \frac{{ - p_{Aa} \left( {p_{AA} - p_{aa} } \right)}}{\beta }\left[ { - \left( {p_{AA} - p_{aa} } \right)} \right] \\ =& \frac{{\beta - \beta p_{Aa} - p_{Aa} \left[ {\left( {p_{AA} - p_{aa} } \right)^{2} } \right]}}{\beta } \\ =& \frac{{\beta - p_{Aa} \left[ {\beta + \left( {p_{AA} - p_{aa} } \right)^{2} } \right]}}{\beta } \\ =& \frac{{\beta - p_{Aa} \left[ {\left( {p_{AA} + p_{aa} } \right)} \right]}}{\beta } \\ =& \frac{{p_{AA} + p_{aa} - \left[ {\left( {p_{AA} - p_{aa} } \right)} \right]^{2} - p_{Aa} \left[ {\left( {p_{AA} + p_{aa} } \right)} \right]}}{\beta } \\ =& \frac{{\left( {1 - p_{Aa} } \right)(p_{AA} + p_{aa} ) - \left[ {\left( {p_{AA} - p_{aa} } \right)} \right]^{2} }}{\beta } \\ =& \frac{{\left[ {\left( {p_{AA} + p_{aa} } \right)} \right]^{2} - \left[ {\left( {p_{AA} - p_{aa} } \right)} \right]^{2} }}{\beta } \\ =& \frac{{4p_{AA} p_{aa} }}{{p_{AA} + p_{aa} - \left[ {\left( {p_{AA} - p_{aa} } \right)} \right]^{2} }} , \\ \end{aligned}$$

which is the same Eq. (11) for genotypes $Aa$ as in the NOIA method.

#### Genotypes $\varvec{aa}$

Equation (15) for individuals with genotypes $aa$ becomes:

$$\begin{aligned} \tau_{D} \left( {aa} \right) = & - p_{Aa} - \frac{{ - p_{Aa} \left( {p_{AA} - p_{aa} } \right)}}{\beta }\left[ { - 1 - \left( {p_{AA} - p_{aa} } \right)} \right] \\ =& - p_{Aa} + \frac{{p_{Aa} \left[ {\left( { - p_{AA} + p_{aa} } \right) - \left( {p_{AA} - p_{aa} } \right)^{2} } \right]}}{\beta } \\ =& - p_{Aa} + \frac{{p_{Aa} \left[ {\left( {p_{AA} + p_{aa} } \right) - \left( {p_{AA} - p_{aa} } \right)^{2} - 2p_{AA} } \right]}}{\beta } \\ =& - p_{Aa} + \frac{{p_{Aa} \left[ {\beta - 2p_{AA} } \right]}}{\beta } \\ =& \frac{{ - p_{Aa} \beta + p_{Aa} \left[ {\beta - 2p_{AA} } \right]}}{\beta } \\ =& \frac{{ - 2p_{AA} p_{Aa} }}{{p_{AA} + p_{aa} - \left[ {\left( {p_{AA} - p_{aa} } \right)} \right]^{2} }} , \\ \end{aligned}$$

which is the same Eq. (11) for genotypes$aa$ as in the NOIA method.
